# Set Protein Is Involved in FLT3 Membrane Trafficking

**DOI:** 10.3390/cancers15082233

**Published:** 2023-04-10

**Authors:** Nerea Marcotegui, Silvia Romero-Murillo, Javier Marco-Sanz, Irene Peris, Blanca S. Berrozpe, Carmen Vicente, María D. Odero, Elena Arriazu

**Affiliations:** 1 Centro de Investigación Médica Aplicada (CIMA), University of Navarra, 31008 Pamplona, Spain; 2 Department of Biochemistry and Genetics, University of Navarra, 31008 Pamplona, Spain; 3 Instituto de Investigación Sanitaria de Navarra (IdiSNA), 31008 Pamplona, Spain; 4 Centro de Investigación Biomédica en Red Cancer (CIBERONC), Instituto de Salud Carlos III, 28029 Madrid, Spain

**Keywords:** AML, FLT3, 3′-UTR, SET/I2PP2A, HuR

## Abstract

**Simple Summary:**

Internal tandem duplication mutations in the FLT3 tyrosine kinase receptor (FLT3-ITD) are associated with poor prognosis in patients with acute myeloid leukemia. These mutations cause constitutive activation of FLT3, altering the underlying signaling pathways and retaining FLT3 in the endoplasmic reticulum (ER). However, the mechanism that determines this peculiar localization is not fully understood. Here, we show that SET acts as a scaffold protein for nascent wild-type FLT3, facilitating its transport to the membrane. By contrast, the FLT3-ITD mutation impairs SET/FLT3 binding, leading to its retention in the ER. Of note, the tyrosine kinase inhibitor midostaurin promotes SET/FLT3 binding, increasing FLT3 in the membrane.

**Abstract:**

The in-frame internal tandem duplication (ITD) of the FLT3 gene is an important negative prognostic factor in acute myeloid leukemia (AML). FLT3-ITD is constitutive active and partially retained in the endoplasmic reticulum (ER). Recent reports show that 3′UTRs function as scaffolds that can regulate the localization of plasma membrane proteins by recruiting the HuR-interacting protein SET to the site of translation. Therefore, we hypothesized that SET could mediate the FLT3 membrane location and that the FLT3-ITD mutation could somehow disrupt the model, impairing its membrane translocation. Immunofluorescence and immunoprecipitation assays demonstrated that SET and FLT3 co-localize and interact in FLT3-WT cells but hardly in FLT3-ITD. SET/FLT3 interaction occurs before FLT3 glycosylation. Furthermore, RNA immunoprecipitation in FLT3-WT cells confirmed that this interaction occurs through the binding of HuR to the 3′UTR of FLT3. HuR inhibition and SET nuclear retention reduced FLT3 in the membrane of FLT3-WT cells, indicating that both proteins are involved in FLT3 membrane trafficking. Interestingly, the FLT3 inhibitor midostaurin increases FLT3 in the membrane and SET/FLT3 binding. Therefore, our results show that SET is involved in the transport of FLT3-WT to the membrane; however, SET barely binds FLT3 in FLT3-ITD cells, contributing to its retention in the ER.

## 1. Introduction

FLT3 is a class III receptor tyrosine kinase that regulates hematopoiesis, which is activated by binding of the fms-related tyrosine kinase 3 ligand (FLT3LG) to the extracellular domain. After activation, homodimers are formed in the plasma membrane, leading to the autophosphorylation of the receptor. The activated receptor kinase phosphorylates and activates diverse cytoplasmic effector molecules involved in apoptosis, proliferation, and differentiation of hematopoietic cells in the bone marrow. In normal cells, the juxtamembrane region has an autoinhibitory role via intramolecular interactions with the catalytic domain [1,2,3]. Approximately one-third of AML patients carry constitutive activating mutations in the FLT3 gene, mostly internal tandem duplication (ITD), which are in-frame duplications of nucleotide sequences with different lengths and insertion sites [4]. The presence of ITDs disrupts the auto-inhibitory effect, resulting in constitutive activation [5]. FLT3 subcellular localization matters for the activation of signaling cascades. FLT3-WT localizes to the membrane and activates pathways such as PI3K/AKT and MAPK/ERK, whereas FLT3-ITD localizes to the endoplasmic reticulum (ER) and activates the STAT5 pathway, repressing C/EBP and Pu.1 [6]. FLT3-ITD AMLs are associated with a higher leukemic burden and an inferior prognosis compared to FLT3-WT AMLs [7,8]. The natural history of FLT3-mutated AML changed after the approval of midostaurin for frontline therapy and gilteritinib for relapsed or refractory patients [9]. Nevertheless, despite initial clinical responses to FLT3 kinase inhibitors, patients eventually relapse. Resistance mechanisms include the acquisition of secondary FLT3 mutations and protective stromal signaling within the bone marrow niche [10]. In the new ELN classification (2022), AMLs with FLT3-ITD are categorized in the intermediate-risk group, irrespective of the allelic ratio or concurrent presence of NPM1 mutation, owing to the impact of midostaurin-based therapy on FLT3-ITD without NPM1 mutation, among other reasons [11].

SET is a multifunctional protein that mediates essential functions in the cell and is overexpressed in ~30% of AML patients [12]. The nucleus-cytoplasm shuttling of SET is controlled by the interaction with exportin CRM1 and by the phosphorylation of serine 9 in one of the SET nuclear localization signals by casein kinase 2 (CK2) [13]. In the cytoplasm, SET can bind to RAC1-GTP (Rac family small GTPase 1) and migrate to the plasma membrane, where it enhances RAC1 signaling pathways thanks to its role as a PP2A inhibitor, facilitating migration [14]. SET is also found in the ER, acting as a suppressor of NM23-H1 by preventing its exonuclease functions [15]. In AML cells, SET is mostly located in a half-moon shape between the nucleus and the cell membrane, where it partly binds PP2A [16]. Recent studies show that 3′UTRs enable the formation of protein complexes by binding mRNA-binding proteins that recruit effector proteins [17]. It has been reported that the 3′UTRs of some genes encoding plasma membrane receptors, such as CD47, act as scaffolds to recruit a protein complex containing the mRNA-binding protein HuR and SET to the translation site. This facilitates the interaction of SET with the newly translated cytoplasmic domains of CD47 and results in subsequent translocation of the protein to the plasma membrane via activated RAC1 [18]. Interestingly, the 3′UTR-mediated interaction of SET with membrane proteins takes place in membraneless organelles called TIS granules, formed by the broadly expressed mRNA-binding protein TIS11B. TIS granules form a reticular meshwork intertwined with the ER that creates a subcellular compartment with a biophysically and biochemically distinct environment from the cytoplasm [19]. Therefore, we hypothesized that SET could mediate the FLT3 membrane location and that the FLT3-ITD mutation could somehow disrupt the model, impairing its membrane translocation. Here we found that SET is involved in the transport of FLT3-WT to the membrane. In FLT3-WT cells, SET and FLT3 interact through the binding of HuR to the 3′UTR of FLT3. This would allow SET to interact with the newly translated FLT3 and to translocate this protein to the plasma membrane via active RAC1. By contrast, in FLT3-ITD cells, SET is barely able to bind to FLT3, contributing to its retention in the ER. Furthermore, our results show that the FLT3 inhibitor midostaurin increases FLT3 in the membrane by promoting SET/FLT3 binding.

## 2. Material and Methods

### 2.1. General Methodology

Details on general methodologies such as western blot, protein immunoprecipitation, RNA extraction, RT-PCR and qPCR have been previously described [12,16]. The reagents and antibodies used are displayed in Table S1 and Table S2, respectively.

### 2.2. Cell Culture and Treatments

HEK-293, HL60, MOLM-13, MV4-11 and OCI-AML3 cell lines were grown and maintained in RPMI-1640 (Invitrogen, Waltham, MA, USA) supplemented with 10% FBS, penicillin G (100 U/mL) and streptomycin (0.1 mg/mL) at 37 °C in a 5% CO_2_ atmosphere. Ba/F3 cells were grown and maintained in RPMI-1640 (Invitrogen) supplemented with 10% FBS, Interleukin-3 (1 ng/mL), penicillin G (100U/mL) and streptomycin (0.1 mg/mL) at 37 °C in a 5% CO_2_ atmosphere. Cell lines were obtained from DSMZ. 24 h prior to treatments, cells were plated at 600,000 cells/mL.

### 2.3. Immunofluorescence

One hundred thousand cells were seeded on coverslips coated with poly-L-lysine (Sigma, St. Louis, MO, USA), fixed with 4% paraformaldehyde (Thermo-Scientific, Waltham, MA, USA) and permeabilized with 0.1% Triton-X-100. After blocking with 5% FBS, incubation with primary and secondary antibodies was performed (Table S2). Images were acquired using a Confocal Scanning Laser Microscopy Zeiss LSM 800 with 63× immersion oil objective. Image quantification was performed using Fiji software [20]. For co-localization, the red, green and red-green co-localization volumes (umÂ^3^) were quantified and referred to total fluorescence and cell volume.

### 2.4. Fluorescence-Activated Cell Sorting (FACS)

After treatments, cells were collected and washed twice with PBS. Then, PE anti-human CD135 (Table S2) 1:10 in 0.5% FBS/PBS was incubated for 30 min at RT in the dark. Finally, cells were washed twice with PBS and resuspended in PBS. For total FLT3, after membrane staining, cells were permeabilized and fixed with Fixation/Permeabilization buffer (eBioscience Invitrogen #00-5123, Waltham, MA, USA) according to manufacturing protocol. Then, cells were washed with permeabilization buffer (#00-8333 Invitrogen) and stained again with PE anti-human CD135, as indicated above. Finally, cells were washed with permeabilization buffer and resuspended in PBS. For negative control, cells were stained with an isotype Antibody. At least 30,000 cells were analyzed on a BD FACS Calibur cell analyzer (BD Biosciences, San Jose, CA, USA), and FACS data were computed using the FlowJo VX software (v10.8).

### 2.5. RNA Immunoprecipitation (RIP)

Twenty million cells were collected for cross-linking with 1% formaldehyde for 15 min in shaking. The cross-linking was stopped by incubating with glycine 0.25 M for 5 min. Pellet was then resuspended in 1ml lysis buffer (50 mM TRIS/HCl, pH 7.4, 100 mM NaCl, 0.5% Triton X-100, 5 mM EDTA, 0.25% Na-deoxycholate, Protease Inhibitor (Roche), RNase Inhibitor (NEB)) and sonicated for 3 cycles of 10 s using Picoruptor (Diagenode, Ougrée, Belgium). Samples were centrifuged for 15 min at 16,000× *g*, and the supernatants were collected. The extract was pre-cleared with 50 µL of pre-washed Protein A/G magnetic Dynabeads (Thermo Fisher, Madrid, Spain). At this point, 10% of the sample was separated as inputs (RNA and proteins). Then, 10 µg of ELAV1/HuR or rabbit IgG antibody was added and incubated overnight at 4 °C in rotation. 50 µL of pre-washed Protein A/G magnetic Dynabeads was added to each tube and incubated for 2 h at 4 °C in rotation. Beads were washed once with lysis buffer and 5 times with high-salt buffer (50 mM TRIS/HCl pH 7.4, 1 M NaCl, 0.5% Triton X-100, 1 M Urea, 5 mM EDTA, 1 mM DTT). After the last wash, the beads were resuspended in 100 μL of RIP buffer (50 mM HEPES pH 7.5, 0.1 M NaCl, 5 mM EDTA, 10 mM DTT, 0.5% Triton X-100, 1% SDS) and incubated for 45 min at 70 °C to revert crosslinking. Finally, the samples were treated with proteinase K (Invitrogen, Thermo Fisher) for 20 min at 37 °C and proceeded to RNA extraction with TRIzol reagent (Invitrogen, Thermo Fisher) to extract RNA. For proteins, beads and input were resuspended in 1× Protein loading buffer (BioRad, Madrid, Spain) followed by Western Blot.

### 2.6. siRNA Transfection

siRNAs were from Ambion. For the ELAVL1 (HUR) gene we used siELAV1-1 (invitrogen #s4609) sense 5′-UGAACUACGUGACCGCGAAtt-3′, antisense 5′-UUCGCGGUCACGUAGUUCAca-3′; siELAV1-2 (invitrogen #s4610) sense 5′-GCGUUUAUCCGGUUUGACAtt-3′, antisense 5′-UGUCAAACCGGAUAAACGCaa-3′. For silencing experiments, 2,000,000 cells were transfected with 400 nM of siRNA using GenePulser XcellTM (Bio-Rad, Madrid, Spain) with 300 V and 1000 μF and incubated for 48 h.

### 2.7. Ectopic Expression of FLT3-WT and FLT3-ITD in Ba/F3 Cells

Plasmids MSCV-Neo-FLT3 (transcript variant 1, NM_004119.3) and MSCV-Neo-FLT3-ITD (transcript variant 1, NM_004119.3 with 21 bp insertion 3′- GAGAATATGAATATGATCTCA-5′) were kindly provided by Dr. Cools (Belgium). Lentiviral particles were produced in HEK-293 cells. For transfection into HEK-293 cells, Genejuice Transfection reagent (Novagen #70967, Darmstadt, Alemania) was used according to the manufacturer’s protocol. To account for differences in the sizes of transfected plasmids, the same molar amounts were transfected. After transfection, cells were incubated for 18 h at 37 °C (5%CO_2_), when the medium was replaced by fresh RPMI 10% FBS and incubated for 24 h more. After that time, the filtered medium from infected HEK-293 was added to Ba/F3 cells in the presence of 8 µg/mL Polibrene (Santa Cruz Biotechnologies, sc-134220, Heidelberg. Germany) and incubated for 24 h. Then, replaced with fresh medium (RPMI 10% FBS + mIL-3) and incubated for 72 h at 37 °C (5%CO_2_). When selecting the infected cells, the cells were grown in the presence of high amounts of neomycin (1300 µg/mL) for 1 week (by changing the medium every 2 days), and western-blot and PCR analyses were performed to confirm that FLT3- WT and FLT3-ITD were ectopically expressed.

### 2.8. Statistical Analysis

The data represented are the means of 3 independent experiments. Statistical comparisons were carried out using the non-parametric method Kruskal–Wallis test for more than 2 independent samples, followed by Mann–Whitney U test to compare the 2 groups when the distribution was not normal (Shapiro–Wilk test *p* < 0.05). Significance was considered when *p* < 0.05.

## 3. Results

### 3.1. FLT3 Interacts with SET in FLT3-WT Cells, but Not in FLT3-ITD Cells

**Figure 1:**
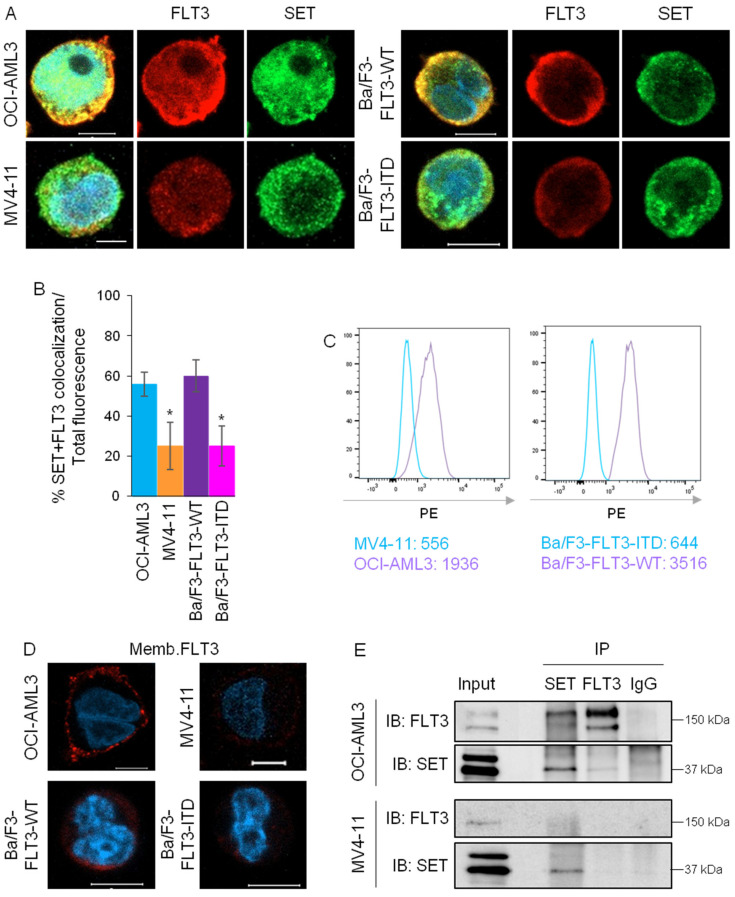
SET and FLT3 interact in FLT3-WT but not in FLT3-ITD AML cells. (**A**) Immunofluorescence of SET (green) and FLT3 (red) in AML cell lines OCI-AML3 (FLT3-WT) and MV4-11 (FLT3-ITD) and Ba/F3 cells overexpressing FLT3-WT or FLT3-ITD. Confocal microscopy showed co-localization (yellow) of both proteins in FLT3-WT cells and hardly in FLT3-ITD cells. Nuclei are stained with DAPI (blue). The scale bar represents 5 µm. (**B**) Bar graphs representing quantification of mean values ± SD of SET and FLT3 co-localization referred to total cell volume, showing significantly less co-localization of SET/FLT3 in FLT3-ITD cells compared to FLT3-WT cells. (**C**) FACS analysis of membrane FLT3 in AML cells without permeabilization. FLT3-WT cells have more surface FLT3 compared to FLT3-ITD cells. Numbers represent mean PE fluorescence intensity. Representative image from more than four experiments. (**D**) Fluorescence confocal microscopy of endogenous FLT3 (red) in cells without permeabilization, where FLT3 surface expression is demonstrated in FLT3-WT cells but barely in FLT3-ITD cells. Cell nuclei are stained with DAPI (blue). Scale bar represents 5 µm (**E**) Immunoprecipitation of SET and FLT3 in AML cells and subsequent western blot analysis, showing protein-protein interaction between SET/FLT3. Representative WBs are shown. * *p* < 0.05 FLT3-ITD cells vs. FLT3-WT cells. The uncropped bolts are shown in Supplementary Materials File S1.

As expected, SET localizes to the nucleus and ER of AML cells but not to other organelles (Figure S1A,B). Immunofluorescence analysis by confocal microscopy showed that SET and FLT3 co-localize in a near-membrane region in FLT3-WT cells (HL60 and OCI-AML3) but not in FLT3-ITD cells (MV4-11, homozygous for FLT3-ITD, and MOLM-13, heterozygous for FLT3-ITD) (Figure 1A,B and Figures S2 and S7A). This was confirmed in Ba/F3 cells overexpressing either FLT3-WT or FLT3-ITD (Figure 1A,B and Figure S7A). FACS analysis and immunofluorescence of non-permeabilized cells confirmed that FLT3-WT cells had significantly more FLT3 in the membrane than FLT3-ITD cells (Figure 1C,D). Since MOLM-13 cells are heterozygous for FLT3-ITD, we used OCI-AML3 (FLT3-WT) and MV4-11 cells (homozygous for FLT3-ITD) for further experiments. Immunoprecipitation assays showed that the interaction between SET and FLT3 was much higher in FLT3-WT cells than in FLT3-ITD cells (Figure 1E). Taken together, our results show that FLT3 binds to SET in FLT3-WT cells, whereas the SET/FLT3 interaction is hardly found in FLT3-ITD cells.

### 3.2. FLT3-WT mRNA Forms a Complex with HuR and SET

**Figure 2:**
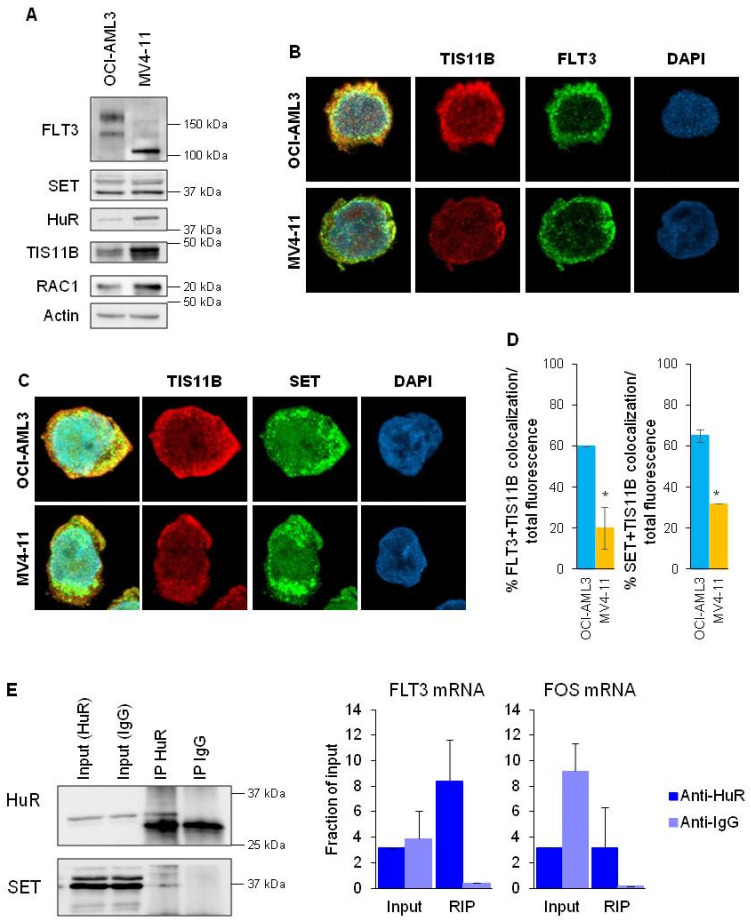
FLT3, TIS11B, HuR and SET form a complex in FLT3-WT cells. (**A**) Western blot analysis of OCI-AML3 (FLT3-WT) and MV4-11 (FLT3-ITD) for FLT3, SET, HuR, TIS11B, RAC1, and normalized with β-Actin, indicating expression of those proteins in both cell lines. Representative WBs are shown. (**B**) Immunofluorescence of FLT3 (green) and TIS11B (red) in AML cell lines OCI-AML3 (FLT3-WT) and MV4-11 (FLT3-ITD). The confocal microscopy pictures showed co-localization (yellow) of both proteins in FLT3-WT cells and hardly in FLT3-ITD cells. Nuclei are stained with DAPI (blue). (**C**) Immunofluorescence of SET (green) and TIS11B (red) in AML cell lines OCI-AML3 (FLT3-WT) and MV4-11 (FLT3-ITD). Confocal microscopy showed co-localization (yellow) of both proteins in FLT3-WT cells and hardly in FLT3-ITD cells. Nuclei are stained with DAPI (blue). (**D**) Co-localization analysis of FLT3 and TIS11B and SET and TIS11B in OCI-AML3 and MV4-11. Mean values ± SEM are represented. (**E**) RNA-immunoprecipitation in OCI-AML3 cells. Protein–RNA complexes were pulled down with anti-HuR antibody, and FLT3 abundance was normalized to HPRT and is shown as a fraction of input. Left, analysis by western blot of immunoprecipitated lysates, showing immunoprecipitation of HuR along with co-immunoprecipitation of SET. Right, RT-qPCR analysis of FLT3 mRNA immunoprecipitated along with the positive control FOS. * *p* < 0.05 FLT3-ITD cells vs. FLT3-WT cells. Protein–RNA complexes were pulled down with anti-HuR antibody, and GFP abundance was normalized to HPRT and is shown as a fraction of input. The uncropped bolts are shown in Supplementary Materials File S1.

mRNAs attracted to the TIS organelle have been reported to have multiple AU-rich element (ARE) motifs in their 3′UTR [19]. The analysis of the 3′UTR sequences of FLT3 showed that it contains AREs motifs for HuR binding and the TIS11B canonical ARE AUUUA (Figure S4). Besides, the FLT3 protein has a transmembrane domain and, in the cytoplasmic area, has enrichment regions with positive amino acids (arginine, R and lysine, K) that would allow SET binding (Figure S5). Therefore, FLT3 accomplishes the requirements for being translated into the TIS granules. We first analyzed the expression of those proteins in both FLT3-WT and FLT3-ITD cells. All cell lines express the TIS11B and HuR proteins, along with FLT3, SET and RAC1 (Figure 2A and Figure S3A). In FLT3-WT cells, there was co-localization between FLT3 and TIS11B (Figure 2B,D and Figures S3C and S7B) and SET and TIS11B (Figure 2C,D and Figures S3D and S7B). However, in FLT3-ITD cells, the co-localization between FLT3 and TIS11B was diminished (Figure 2D and Figures S3C,D and S7B). Thus, our results suggest that SET and FLT3 are associated with TIS11B in FLT3-WT cells and, to a lesser extent, in FLT3-ITD cells. If FLT3-WT is translated in the TIS granules and SET binds to the nascent FLT3 protein, the HuR mRNA binding protein should bind to the 3′UTR of FLT3. To prove that, RNA immunoprecipitation (RIP) was performed in FLT3-WT cells. HuR bound to FLT3 mRNA and to the positive control FOS mRNA and co-immunoprecipitated with SET (Figure 2E), suggesting that the three proteins form a complex in FLT3-WT cells. Then, our results show that the SET/FLT3 protein interaction is mediated by TIS11B and HuR.

### 3.3. SET Modulates FLT3-WT Membrane Transport

**Figure 3:**
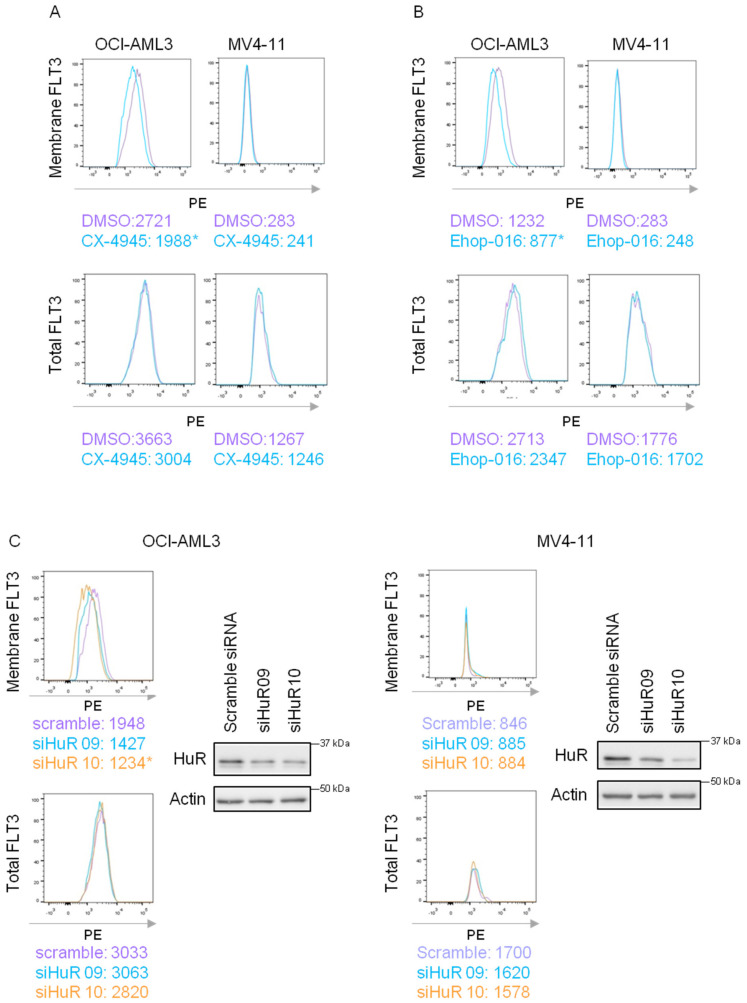
HuR/SET/RAC1 complex regulates FLT3 membrane translocation. (**A**) AML cells OCI-AML3 (FLT3-WT) and MV4-11 (FLT3-ITD) were treated with the CK2 inhibitor CX-4945 (5 µM, 24 h). FACS analysis of membrane FLT3 in non-permeabilized and total FLT3 in permeabilized cells showed a decrease in membrane FLT3, without changes in total FLT3, after CK2 inhibition due to SET nuclear retention in FLT3-WT. This was not observed in FLT3-ITD. Numbers represent mean PE fluorescence intensity. Representative image from more than 6 experiments is shown. (**B**) AML cells OCI-AML3 (FLT3-WT) and MV4-11 (FLT3-ITD) were treated with the RAC1 inhibitor EHop-016 under IC50 values (2.5 µM for MV4-11 and 4 µM for OCI-AML3, 24 h) and then FACS analysis of membrane FLT3 in non-permeabilized and total FLT3 in permeabilized cells was performed. The experiment showed a decrease in membrane FLT3-WT without changes in total FLT3 after RAC1 inhibition. This was not observed in FLT3-ITD. Numbers represent mean PE fluorescence intensity. Representative image from more than 6 experiments is shown. (**C**) Knockdown of ELAV1/HuR with siRNA (400 nM for 48 h), using scramble siRNA as control, in OCI-AML3 and MV4-11 cells. FACS analyses of membrane FLT3 in non-permeabilized and total FLT3 in permeabilized cells were performed, indicating less membrane FLT3, without changes in total FLT3, after HuR silencing in FLT3-WT cells. This was not observed in FLT3-ITD cells. Numbers represent mean PE fluorescence intensity. Representative image from more than six experiments is shown. Western blot analysis of HuR for checking the silencing compares to the internal control β-Actin. Representative WBs are shown (* *p* < 0.05). The uncropped bolts are shown in Supplementary Materials File S1.

Next, we analyzed whether SET was regulating FLT3 translocation to the membrane. It was not possible to maintain SET knockdown in our cells by CRISPR-Cas9, shRNA or siRNA: our results suggest that AML cell lines could be dependent on SET high expression levels for sustained oncogenic growth and that SET knockout could seriously affect their viability and limit the number of cells available for further experiments). Therefore, we modulated SET trafficking by treating cells with the CK2 inhibitor CX-4945, which retains SET in the nucleus [16]. FACS analysis showed that CX-4945 treatment decreased FLT3 in the membrane of FLT3-WT cells without altering FLT3 total levels and had almost no effect on FLT3-ITD cells (Figure 3A and Figure S8A). SET binds to the GTPase RAC1 to allow protein translocation [14]; thus, we next determined whether RAC1 inhibition also modulated FLT3 at the membrane. Treatment with the RAC1 inhibitor EHop-016 decreased FLT3 in the membrane of FLT3-WT cells without altering FLT3 total protein and barely in FLT3-ITD cells (Figure 3B and Figure S8B). On the other hand, the binding of HuR to the FLT3 3′UTR is necessary for the binding of SET and, consequently, for the transport of FLT3 to the membrane. FACS analysis revealed that HuR silencing decreased FLT3 in the membrane of FLT3-WT without modulating total FLT3 and hardly in FLT3-ITD AML cells (Figure 3C and Figure S8C). Taken together, our results suggest that the complex SET/RAC1/HuR is involved in the FLT3 transport to the cytoplasmic membrane.

### 3.4. SET Binds to FLT3 Underglycosylated in the ER

**Figure 4:**
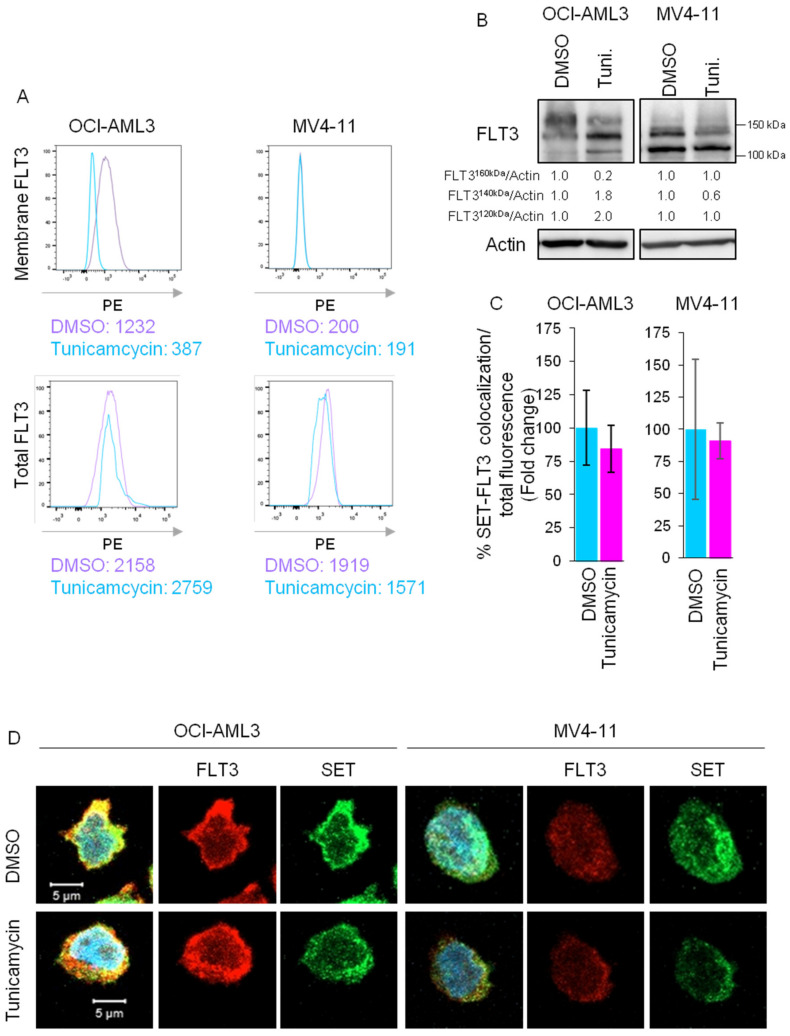
Glycosylation inhibition decreases FLT3 in the membrane but does not disrupt SET/FLT3 interaction in FLT3-WT AML cells. AML cell lines OCI-AML3 (FLT3-WT) and MV4-11 (FLT3-ITD) were treated with tunicamycin (200 µg/mL) for 24 h (**A**) FACS analysis of membrane FLT3 in AML cells without permeabilization and total FLT3 in permeabilized cells. Inhibition of the glycosylation decreased membrane FLT3 in FLT3-WT but not in FLT3-ITD cells without changing total FLT3 levels. Numbers represent mean PE fluorescence intensity. Representative image from more than 4 experiments is shown. (**B**) Western blot analysis of FLT3 after tunicamycin treatment in OCI-AML3 and MV4-11 cells. Quantification of the relative FLT3 bands demonstrated a decrease in the 150 kDa mature band along with an increase in <150 kDa immature bands in FLT3-WT cells but not in FLT3-ITD cells. A representative western blot is shown. (**C**) Bar graphs representing quantification of Mean values ± SD of SET and FLT3 co-localization referred to total cell volume, demonstrating that SET/FLT3 interaction is not disrupted after glycosylation inhibition. (**D**) Immunofluorescence of SET (green) and FLT3 (red) in AML cell lines. Confocal microscopy showed co-localization (yellow) of both proteins even after glycosylation inhibition. Nuclei are stained with DAPI (blue). The scale bar represents 5 µm. The uncropped bolts are shown in Supplementary Materials File S1.

FLT3-WT is synthesized as a 130 kDa underglycosylated species and then is folded in the ER and exported to the Golgi apparatus, where it is glycosylated to form a 150 kDa protein prior to translocation to the cell surface [21,22]. By contrast, FLT3-ITD is partially retained in the ER as the underglycosylated 130 kDa species [21]. We, therefore, speculate that if SET binds to the nascent FLT3 protein prior to membrane transport and glycosylation is required for membrane transport, the SET/FLT3 interaction would be maintained even if FLT3 is not glycosylated. To demonstrate that, FLT3-WT cells were treated with tunicamycin, which inhibits glycosylation of proteins leading to unfolded protein response (UPR). FACS analysis revealed that tunicamycin treatment significantly decreased FLT3 in the membrane without altering total FLT3 levels (Figure 4A), correlating with a decrease in 150 kDa-mature FLT3 band (Figure 4B) without altering SET/FLT3 co-localization (Figure 4C,D and Figure S7C), suggesting that SET/FLT3 association occurs prior to glycosylation. Furthermore, inhibition of glycosylation increased UPR markers (Figure S6) due to protein accumulation in the ER.

### 3.5. FLT3 Inactivation Enhances SET/FLT3 Binding in FLT3-ITD Cells

**Figure 5:**
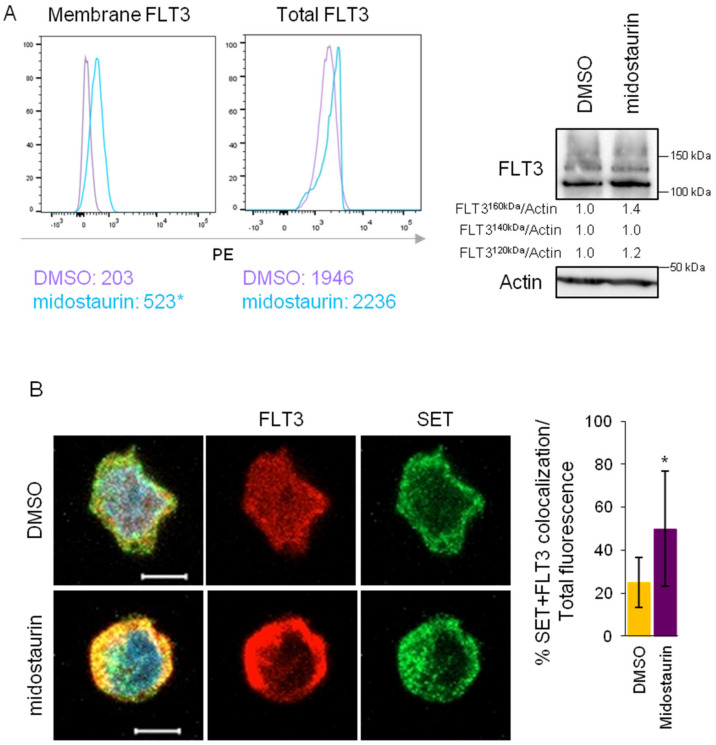
The tyrosine kinase inhibitor midostaurin enhances SET/FLT3 binding and FLT3 transport to the membrane in FLT3-ITD cells. AML cell line MV4-11 (FLT3-ITD) was treated with midostaurin (20 nM) for 24 h (**A**) FACS analysis of membrane FLT3 in MV4-11 cells without permeabilization, and total FLT3 in permeabilized cells showed increased membrane FLT3 after midostaurin treatment with a slight increase in total FLT3 which did not disrupt the significant increase of membrane FLT3 after midostaurin treatment. Numbers represent mean PE fluorescence intensity. Representative image from more than four experiments is shown. Western blot analysis of FLT3 after midostaurin treatment in OCI-AML3 and MV4-11 cells. Quantification of the relative FLT3 bands demonstrated a decrease in the 150 kDa mature band along with an increase in <150 kDa immature bands in FLT3-WT cells but not in FLT3-ITD cells. A representative western blot is shown. (**B**) Immunofluorescence of SET (green) and FLT3 (red) in MV4-11 cell lines. Confocal microscopy pictures showed increased co-localization (yellow) of both proteins after midostaurin treatment. Nuclei are stained with DAPI (blue). The scale bar represents 5 µm. The bar graph represents mean values±SD of SET and FLT3 co-localization referred to as total fluorescence. * *p* < 0.05. The uncropped bolts are shown in Supplementary Materials File S1.

Previous studies have demonstrated that treatment of AML FLT3-ITD cells with tyrosine kinase inhibitors increases FLT3 in the membrane [23], although the precise mechanism remains to be defined. We, therefore, hypothesize that if SET mediates FLT3 trafficking, treatment of FLT3-ITD cells with FLT3 inhibitors might also modulate the SET/FLT3 association. To prove that, MV4-11 cells were treated with midostaurin [24], and FLT3 and SET association and location were analyzed. FACS analysis after midostaurin treatment revealed an increase of FLT3 in the membrane (Figure 5A) that was accompanied by an increase in SET/FLT3 colocalization (Figure 5B). Besides, midostaurin treatment increased total FLT3 at mRNA (1.67-fold change increased compared to the control) and protein levels without altering the fold change increase in FLT3/SET co-localization levels observed (Figure 5). These results suggest that midostaurin promotes FLT3 transport to the cytoplasmic membrane in part by facilitating FLT3 binding to SET.

## 4. Discussion

Internal tandem duplication mutations in the FLT3 tyrosine kinase receptor cause constitutive activation of FLT3, altering the underlying signaling pathways and retaining FLT3 in the ER. However, the mechanism that determines this peculiar localization is not fully understood. Here, we show for the first time that SET acts as a scaffold protein for nascent FLT3, which help FLT3 transport to the membrane. By contrast, the FLT3-ITD mutation impairs the SET/FLT3 binding, leading to its retention in the ER. Of note, the TK inhibitor midostaurin increases FLT3 in the membrane by promoting SET/FLT3 binding.

**Figure 6:**
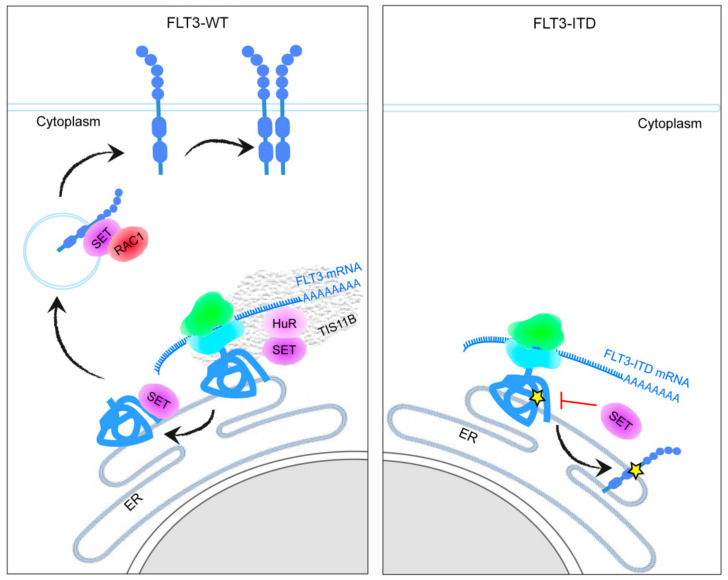
Schematic representation of FLT3-WT transport to the plasma membrane. FLT3-WT mRNA is transcribed in a membraneless organelle made from the mRNA-binding protein TIS11B, which forms a reticular meshwork intertwined with the ER that creates a subcellular compartment with a biophysically and biochemically distinct environment from the cytoplasm. There, the mRNA binding protein HuR binds to the 3′UTR of the FLT3 mRNA and recruits SET. Then, SET binds to the nascent FLT3 protein, which helps translocate FLT3 to the plasma membrane via active RAC1. When FLT3 carries the FLT3-ITD mutation, SET is not able to bind to the nascent protein, contributing to endoplasmic reticulum (ER) retention.

Recent reports show that 3′UTRs function as scaffolds that can regulate the localization of plasma membrane proteins by recruiting the HuR-interacting protein SET to the site of translation [25,26]. In this study, we demonstrate that SET mediates FLT3 localization to the membrane (Figure 6). Immunofluorescence and immunoprecipitation assays demonstrated that SET and FLT3 interact in FLT3-WT cells; by contrast, SET can hardly bind to FLT3 in FLT3-ITD cells. The FLT3-ITD mutation alters the conformation of the protein; therefore, SET could not bind to the nascent FLT3-ITD protein, contributing to its retention in the ER. Another reason could be that the 3′UTRs of the FLT3-WT and FLT3-ITD mRNAs differ; however, in Ba/F3-FLT3 and -FLT3-ITD cells, the overexpressed plasmids do not contain 3′UTRs and even so, we observed less association between SET/FLT3-ITD than between SET/FLT3-WT. Furthermore, RNA immunoprecipitation confirmed that the SET/FLT3-WT interaction occurs through the binding of HuR to the 3′UTR of FLT3 in the same way as described in other genes encoding plasma membrane receptors [17].

Although the aberrant subcellular location of FLT3-ITD in AML cells and the subsequent activation signaling pathways is well known, the underlying mechanisms are poorly known. It has been reported that apart from glycosylation, other FLT3 post-translation modifications can regulate FLT3 location [27,28]. Our study adds to these findings that FLT3 location modulation starts at the mRNA translation level. In the presence of the mRNA binding protein TIS11B, which forms the precise niche, the mRNA binding protein HuR binds to the 3′UTR-FLT3 facilitating the recruitment of SET, which acts as FLT3 scaffold nascent protein (Figure 6). Then, the complex SET/FLT3 recruits the GTPase RAC1, which facilitates the transport to the cell membrane, thanks to the PP2A inhibitory activity of SET [14].

Post-translational modifications are imperative for the correct folding and function of proteins. It is well known that glycosylation is a necessary step for the plasma membrane location of FLT3 [21,22]. Recent studies hint that glycosylation differs in the WT- and ITD-FLT3 proteins [29,30]; however, our results suggest a more complex scenario since SET/FLT3 binding is prior to glycosylation. However, FLT3-SET-RAC1 binding is not sufficient for correct membrane transport because glycosylation inhibition impaired FLT3 membrane transport. These results corroborate that SET acts as a scaffolding protein for the nascent FLT3 protein and that it is necessary for the recruitment of RAC1, facilitating the transport of FLT3 to the membrane.

## 5. Conclusions

In conclusion, our study describes a new mechanism of FLT3 transport (Figure 6). In FLT3-WT cells, SET and FLT3 interact through the binding of HuR to the 3′UTR of FLT3. This allows SET to interact with the newly translated FLT3 and to translocate this protein to the plasma membrane via active RAC1, which provides the GTPase activity needed. By contrast, in FLT3-ITD cells, SET is barely able to bind to FLT3, contributing to its retention in the ER. Furthermore, our results show that the FLT3 inhibitor midostaurin promotes FLT3 transport to the cytoplasmic membrane in part by facilitating FLT3 binding to SET.

## Data Availability

The data presented in this study are available in this article.

## Supplementary Materials

The following supporting information can be downloaded at: https://www.mdpi.com/article/10.3390/cancers15082233/s1. Figure S1: SET is associated with the ER in AML cells. Figure S2: SET and FLT3 co-localize in FLT3-WT cells but hardly co-localize in FLT3. ITD AML cells. Figure S3: SET and FLT3 interact in FLT3-WT AML cells but not in FLT3-ITD AML cells. Figure S4: mRNA sequence of FLT3. Figure S5: FLT3 amino acid sequence. Figure S6: Tunicamycin induces UPR response. Figure S7: Panoramic view of immunofluorescence. Figure S8: Relative bands of FLT3. Table S1: List of drugs used and Table S2: List of Antibodies used. File S1: Original Images for Blots.

## Abbreviations

AML	acute myeloid leukemia
AREs	AU-rich elements
CK2	casein kinase 2
ER	endoplasmic reticulum
FLT3	Fms-related tyrosine kinase 3
FLT3LG	Fms-related tyrosine kinase 3-ligand
ITD	internal tandem duplication
PP2A	protein phosphatase 2A
RIP	RNA immunoprecipitation
TKI	tyrosine kinase inhibitors
UPR	unfolded protein response
WB	western blot
WT	wild type

## References

[1] Griffith J., Black J., Faerman C., Swenson L., Wynn M., Lu F., Lippke J., Saxena K. (2004). The structural basis for autoinhibition of FLT3 by the juxtamembrane domain. Mol. Cell.

[2] Heiss E., Masson K., Sundberg C., Pedersen M., Sun J., Bengtsson S., Ronnstrand L. (2006). Identification of Y589 and Y599 in the juxtamembrane domain of Flt3 as ligand-induced autophosphorylation sites involved in binding of Src family kinases and the protein tyrosine phosphatase SHP2. Blood.

[3] Walter M., Lucet I.S., Patel O., Broughton S.E., Bamert R., Williams N.K., Fantino E., Wilks A.F., Rossjohn J. (2007). The 2.7 A crystal structure of the autoinhibited human c-Fms kinase domain. J. Mol. Biol..

[4] Stirewalt D.L., Kopecky K.J., Meshinchi S., Engel J.H., Pogosova-Agadjanyan E.L., Linsley J., Slovak M.L., Willman C.L., Radich J.P. (2006). Size of FLT3 internal tandem duplication has prognostic significance in patients with acute myeloid leukemia. Blood.

[5] Schnittger S., Bacher U., Haferlach C., Alpermann T., Kern W., Haferlach T. (2012). Diversity of the juxtamembrane and TKD1 mutations (exons 13–15) in the FLT3 gene with regards to mutant load, sequence, length, localization, and correlation with biological data. Genes Chromosomes Cancer.

[6] Choudhary C., Schwable J., Brandts C., Tickenbrock L., Sargin B., Kindler T., Fischer T., Berdel W.E., Muller-Tidow C., Serve H. (2005). AML-associated Flt3 kinase domain mutations show signal transduction differences compared with Flt3 ITD mutations. Blood.

[7] Kantarjian H., Kadia T., DiNardo C., Daver N., Borthakur G., Jabbour E., Garcia-Manero G., Konopleva M., Ravandi F. (2021). Acute myeloid leukemia: Current progress and future directions. Blood Cancer J..

[8] Döhner H., Wei A.H., Löwenberg B. (2021). Towards precision medicine for AML. Nat. Rev. Clin. Oncol..

[9] Perl A.E. (2019). Availability of FLT3 inhibitors: How do we use them?. Blood.

[10] Smith C.C. (2019). The growing landscape of FLT3 inhibition in AML. Hematol. Am. Soc. Hematol. Educ. Program.

[11] Döhner H., Wei A.H., Appelbaum F.R., Craddock C., DiNardo C.D., Dombret H., Ebert B.L., Fenaux P., Godley L.A., Hasserjian R.P. (2022). Diagnosis and management of AML in adults: 2022 recommendations from an international expert panel on behalf of the ELN. Blood.

[12] Cristóbal I., Garcia-Orti L., Cirauqui C., Cortes-Lavaud X., García-Sánchez M.A., Calasanz M.J., Odero M.D. (2012). Overexpression of SET is a recurrent event associated with poor outcome and contributes to protein phosphatase 2A inhibition in acute myeloid leukemia. Haematologica.

[13] Yu G., Yan T., Feng Y., Liu X., Xia Y., Luo H., Wang J.Z., Wang X. (2013). Ser9 phosphorylation causes cytoplasmic detention of I2PP2A/SET in Alzheimer disease. Neurobiol. Aging.

[14] Ten Klooster J.P., Leeuwen I.V., Scheres N., Anthony E.C., Hordijk P.L. (2007). Rac1-induced cell migration requires membrane recruitment of the nuclear oncogene SET. EMBO J..

[15] Fan Z., Beresford P.J., Oh D.Y., Zhang D., Lieberman J. (2003). Tumor suppressor NM23-H1 is a granzyme A-activated DNase during CTL-mediated apoptosis, and the nucleosome assembly protein SET is its inhibitor. Cell.

[16] Arriazu E., Vicente C., Pippa R., Peris I., Martínez-Balsalobre E., García-Ramírez P., Marcotegui N., Igea A., Alignani D., Rifón J. (2020). A new regulatory mechanism of protein phosphatase 2A activity via SET in acute myeloid leukemia. Blood Cancer J..

[17] Mayr C. (2017). Regulation by 3′-Untranslated Regions. Annu. Rev. Genet..

[18] Berkovits B.D., Mayr C. (2015). Alternative 3′UTRs act as scaffolds to regulate membrane protein localization. Nature.

[19] Ma W., Mayr C. (2018). A Membraneless Organelle Associated with the Endoplasmic Reticulum Enables 3′UTR-Mediated Protein-Protein Interactions. Cell.

[20] Schindelin J., Arganda-Carreras I., Frise E., Kaynig V., Longair M., Pietzsch T., Preibisch S., Rueden C., Saalfeld S., Schmid B. (2012). Fiji: An open-source platform for biological-image analysis. Nat. Methods.

[21] Schmidt-Arras D.E., Bohmer A., Markova B., Choudhary C., Serve H., Bohmer F.D. (2005). Tyrosine phosphorylation regulates maturation of receptor tyrosine kinases. Mol. Cell. Biol..

[22] Schmidt-Arras D., Böhmer S.A., Koch S., Müller J.P., Blei L., Cornils H., Bauer R., Korasikha S., Thiede C., Böhmer F.D. (2009). Anchoring of FLT3 in the endoplasmic reticulum alters signaling quality. Blood.

[23] Reiter K., Polzer H., Krupka C., Maiser A., Vick B., Rothenberg-Thurley M., Metzeler K.H., Dörfel D., Salih H.R., Jung G. (2018). Tyrosine kinase inhibition increases the cell surface localization of FLT3-ITD and enhances FLT3-directed immunotherapy of acute myeloid leukemia. Leukemia.

[24] DiNardo C.D., Wei A.H. (2020). How I treat acute myeloid leukemia in the era of new drugs. Blood.

[25] Mayr C. (2019). What Are 3′UTRs Doing?. Cold Spring Harb. Perspect. Biol..

[26] Lee S.H., Mayr C. (2019). Gain of Additional BIRC3 Protein Functions through 3′-UTR-Mediated Protein Complex Formation. Mol. Cell.

[27] Lv K., Ren J.G., Han X., Gui J., Gong C., Tong W. (2021). Depalmitoylation rewires FLT3-ITD signaling and exacerbates leukemia progression. Blood.

[28] He X., Zhu Y., Lin Y.C., Li M., Du J., Dong H., Sun J., Zhu L., Wang H., Ding Z. (2019). PRMT1-mediated FLT3 arginine methylation promotes maintenance of FLT3-ITD+ acute myeloid leukemia. Blood.

[29] Duan C., Fukuda T., Isaji T., Qi F., Yang J., Wang Y., Takahashi S., Gu J. (2020). Deficiency of core fucosylation activates cellular signaling dependent on FLT3 expression in a Ba/F3 cell system. FASEB J..

[30] Hu X., Chen F. (2019). Targeting on glycosylation of mutant FLT3 in acute myeloid leukemia. Hematology.

