# Supplementary material for: Set Protein Is Involved in FLT3 Membrane Trafficking
Source: Cancers (Basel). 2023 Apr 10;15(8):2233. doi: 10.3390/cancers15082233 (PMC10137103; doi:10.3390/cancers15082233)
Supplement: Supplementary file 1 [file cancers-15-02233-s001.zip › cancers-2279870-supplementary.pdf]

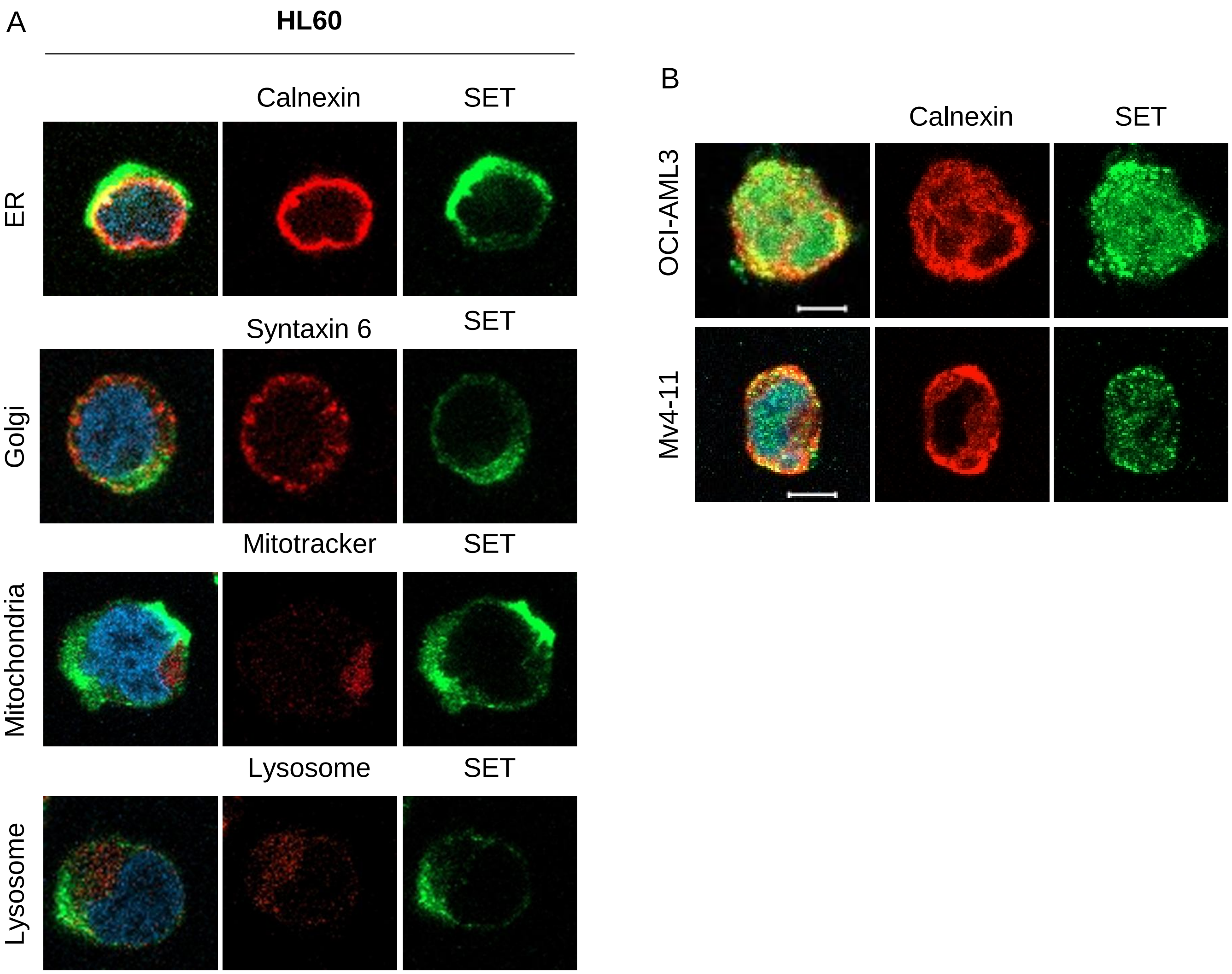

Figure S1. SET is associated to the ER in AML cells.
(A) Immunofluorescence of SET (green) and different organelle markers (red) in HL60 cells. Endoplasmic reticulum (ER) marker Calnexin; Golgi apparatus marker Syntaxin 6; mitochondrial marker mitotracker and lysosomal marker lysotracker. Confocal microscopy showed co-localization (yellow) of the ER marker calnexin and SET. Nucleus are stained with DAPI (blue). (B) Immunofluorescence of SET (green) and the ER marker Calnexin (red) in FLT3-WT AML cells OCI-AML3 and FLT3-ITD cells MV4-11. Representative picture of each staining is shown. Immunofluorescences were visualized by confocal microscopy. Scale bar represent 5 µm.

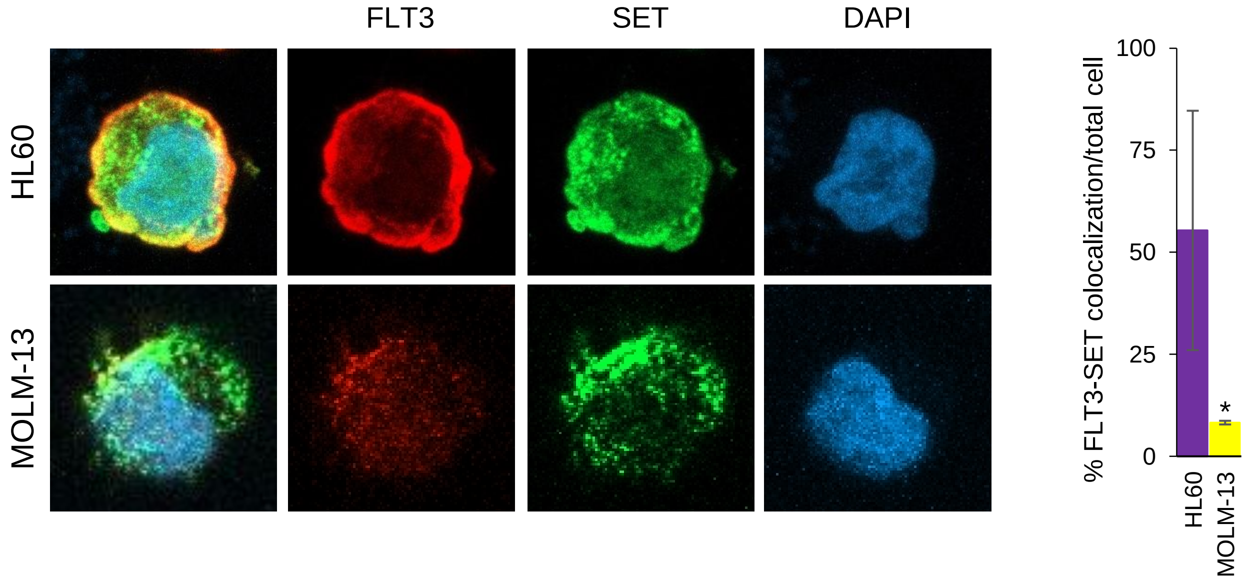

Figure S2. SET and FLT3 co-localize in FLT3-WT cells but hardly co-localize in FLT3-ITD AML cells. Immunofluorescence of SET (green) and FLT3 (red) in AML cell lines HL60 (FLT3-WT) and MOLM-13 (FLT3-ITD). Nuclei were stained with DAPI (blue). Confocal microscopy showed co-localization (yellow) of both protein in FLT3-WT cells and hardly in FLT3-ITD cells. Representative pictures are shown. Immunofluorescences were visualized by confocal microscopy. Scale bars representing quantification of mean values±SD of SET and FLT3 co-localization referred to total cell volume, demonstrating significantly less co-localization of SET/FLT3 in FLT3-ITD cells compare to FLT3-WT cells. The results are expressed as mean values± SEM. Experiments were performed in triplicate four times. *$p<0.05$ FLT3-ITD cells *vs*. FLT3-WT cells.

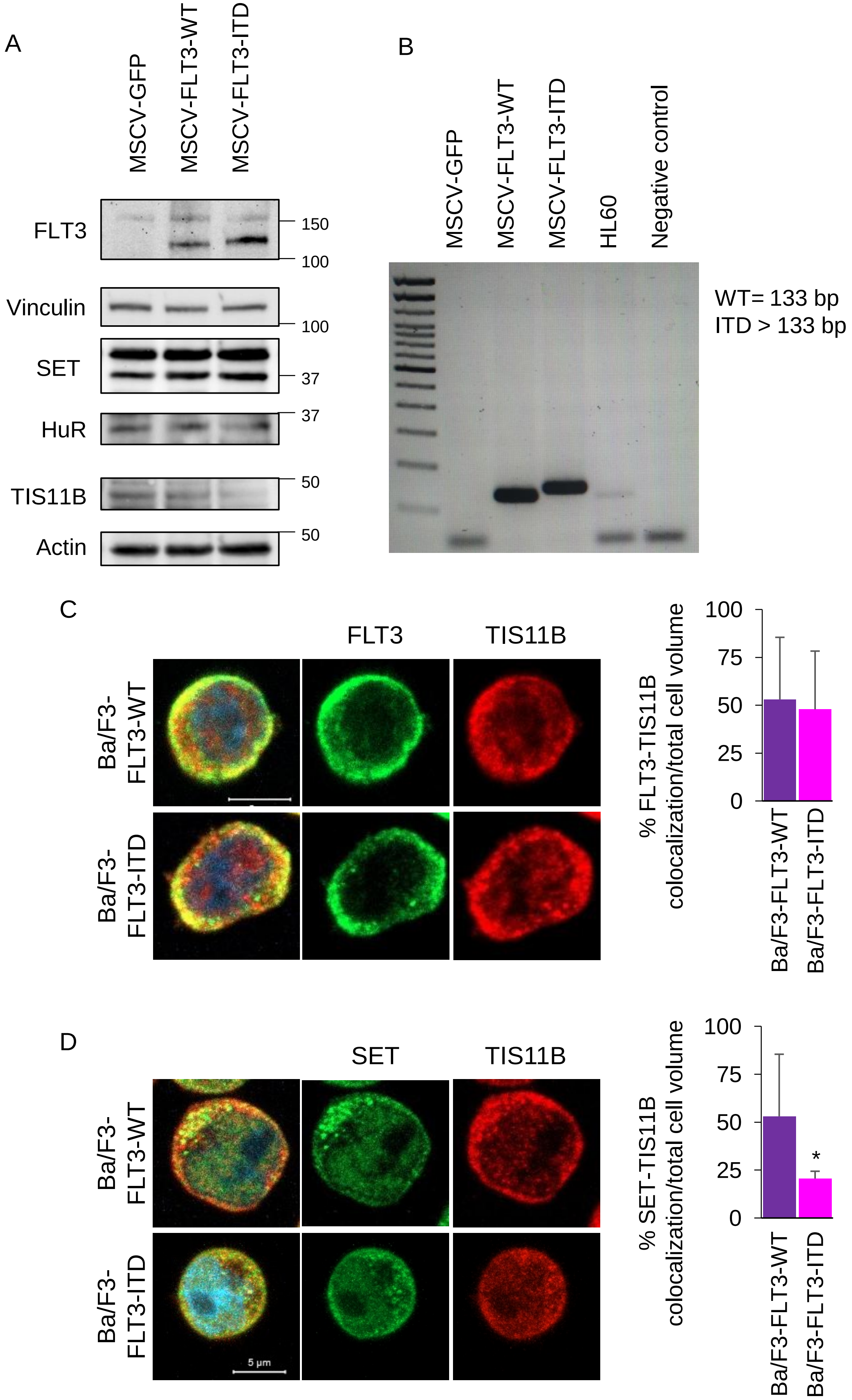
A
MSCV-GFP
MSCV-FLT3-WT
MSCV-FLT3-ITD
FLT3
Vinculin
SET
HuR
TIS11B
Actin
150
100
100
37
37
50
50
B
MSCV-GFP
MSCV-FLT3-WT
MSCV-FLT3-ITD
HL60
Negative control
WT= 133 bp
ITD > 133 bp
C
FLT3
TIS11B
Ba/F3-FLT3-WT
Ba/F3-FLT3-ITD
% FLT3-TIS11B colocalization/total cell volume
100
75
50
25
0
Ba/F3-FLT3-WT
Ba/F3-FLT3-ITD
D
SET
TIS11B
Ba/F3-FLT3-WT
Ba/F3-FLT3-ITD
5 µm
% SET-TIS11B colocalization/total cell volume
100
75
50
25
0
*
Ba/F3-FLT3-WT
Ba/F3-FLT3-ITD

Figure S3. SET and FLT3 interact in FLT3-WT AML cells but not in FLT3-ITD AML cells. (A) Western blot analysis of Ba/F3 over expressing FLT3-WT or FLT3-ITD for FLT3, SET, HuR, TIS11B, RAC1 and normalized with Actin; indicating expression of those proteins in all cell lines. Representative WB are shown. (B) Agarose gel from PCR analysis of DNA from Ba/F3 over expressing the empty vector, FLT3-WT or FLT3-ITD; using the primers Primers: 11F:5´caatttaggtatgaaagcc 3´ and 11R: 5´caaactctaaattttctct 3´. The FLT3-WT product is 133 bp and the FLT3-ITD product is bigger than 133 bp. (C) Immunofluorescence of FLT3 (green) and TIS11B (red) in Ba/F3 over expressing FLT3-WT or FLT3-ITD showed co-localization of both proteins (yellow). (D) Immunofluorescence of SET (green) and TIS11B (red) in Ba/F3 over expressing FLT3-WT or FLT3-ITD showed co-localization of both proteins in FLT3-WT and barely in FLT3-ITD over expressing cells. Nucleus are stained with DAPI (blue). Scale bar represent 5 µm. Graphs represent mean values ± SD of SET and FLT3 co-localization referred to total cell volume.

NM_004119.3 FLT3 [organism=Homo sapiens] [GeneID=2322] [transcript=1]

GCGCCGCTCCAGGCGGCATCGCAGGGCTGGGCCGGCGCGGCCTGGGGACCCCGGGCTCCGGAGGCC**ATG**CCGGCGTTGGCGCGCGAC
GGCGGCCAGCTGCCGCTGCTCGTTGTTTTTTCTGCAATGATATTTGGGACTATTACAAATCAAGATCTGCCTGTGATCAAGTGTGTT
TTAATCAATCATAAGAACAATGATTCATCAGTGGGGAAGTCATCATCATATCCCATGGTATCAGAATCCCCGGAAGACCTCGGGTGT
GCGTTGAGACCCCAGAGCTCAGGGACAGTGTACGAAGCTGCCGCTGTGGAAGTGGATGTATCTGCTTCCATCACACTGCAAGTGCTG
GTCGACGCCCCAGGGAACATTTCCTGTCTCTGGGTCTTTAAGCACAGCTCCCTGAATTGCCAGCCACATTTTGATTTACAAAACAGA
GGAGTTGTTTCCATGGTCATTTTGAAAATGACAGAAACCCAAGCTGGAGAATACCTACTTTTTATTCAGAGTGAAGCTACCAATTAC
ACAATATTGTTTACAGTGAGTATAAGAAATACCCTGCTTTACACATTAAGAAGACCTTACTTTAGAAAAATGGAAAACCAGGACGCC
CTGGTCTGCATATCTGAGAGCGTTCCAGAGCCGATCGTGGAATGGGTGCTTTGCGATTCACAGGGGGAAAGCTGTAAAGAAGAAAGT
CCAGCTGTTGTTAAAAAGGAGGAAAAAGTGCTTCATGAATTATTTGGGACGGACATAAGGTGCTGTGCCAGAAATGAACTGGGCAGG
GAATGCACCAGGCTGTTCACAATAGATCTAAATCAAACTCCTCAGACCACATTGCCACAATTATTTCTTAAAGTAGGGGAACCCTTA
TGGATAAGGTGCAAAGCTGTTCATGTGAACCATGGATTCGGGCTCACCTGGGAATTAGAAAACAAAGCACTCGAGGAGGGCAACTAC
TTTGAGATGAGTACCTATTCAACAAACAGAACTATGATACGGATTCTGTTTGCTTTTGTATCATCAGTGGCAAGAAACGACACCGGA
TACTACACTTGTTCCTCTTCAAAGCATCCCAGTCAATCAGCTTTGGTTACCATCGTAGAAAAGGGATTTATAAATGCTACCAATTCA
AGTGAAGATTATGAAATTGACCAATATGAAGAGTTTTGTTTTTCTGTCAGGTTTAAAGCCTACCCACAAATCAGATGTACGTGGACC
TTCTCTCGAAAATCATTTCCTTGTGAGCAAAAGGGTCTTGATAACGGATACAGCATATCCAAGTTTTGCAATCATAAGCACCAGCCA
GGAGAATATATATTCCATGCAGAAAATGATGATGCCCAATTTACCAAAATGTTCACGCTGAATATAAGAAGGAAACCTCAAGTGCTC
GCAGAAGCATCGGCAAGTCAGGCGTCCTGTTTCTCGGATGGATACCCATTACCATCTTGGACCTGGAAGAAGTGTTCAGACAAGTCT
CCCAACTGCACAGAAGAGATCACAGAAGGAGTCTGGAATAGAAAGGCTAACAGAAAAGTGTTTGGACAGTGGGTGTCGAGCAGTACT
CTAAACATGAGTGAAGCCATAAAAGGGTTCCTGGTCAAGTGCTGTGCATACAATTCCCTTGGCACATCTTGTGAGACGATCCTTTTA
AACTCTCCAGGCCCCTTCCCTTTCATCCAAGACAACATCTCATTCTATGCAACAATTGGTGTTTGTCTCCTCTTCATTGTCGTTTTA
ACCCTGCTAATTTGTCACAAGTACAAAAAGCAATTTAGGTATGAAAGCCAGCTACAGATGGTACAGGTGACCGGCTCCTCAGATAAT
GAGTACTTCTACGTTGATTTCAGAGAATATGAATATGATCTCAAATGGGAGTTTCCAAGAGAAAATTTAGAGTTTGGGAAGGTACTA
GGATCAGGTGCTTTTGGAAAAGTGATGAACGCAACAGCTTATGGAATTAGCAAAACAGGAGTCTCAATCCAGGTTGCCGTCAAAATG
CTGAAAGAAAAAGCAGACAGCTCTGAAAGAGAGGCACTCATGTCAGAACTCAAGATGATGACCCAGCTGGGAAGCCACGAGAATATT
GTGAACCTGCTGGGGGCGTGCACACTGTCAGGACCAATTTACTTGATTTTTGAATACTGTTGCTATGGTGATCTTCTCAACTATCTA
AGAAGTAAAAGAGAAAAATTTCACAGGACTTGGACAGAGATTTTCAAGGAACACAATTTCAGTTTTTACCCCACTTTCCAATCACAT
CCAAATTCCAG**CATGCCTGGTT**CAAGAGAAGTTCAGATACACCCGGACTCGGATCAAATCTCAGGGCTTCATGGGAATTCATTTCAC
TCTGAAGATGAAATTGAATATGAAAACCAAAAAAGGCTGGAAGAAGAGGAGGACTTGAATGTGCTTACATTTGAAGATCTTCTTTGC
TTTGCATATCAAGTTGCCAAAGGAATGGAATTTCTGGAATTTAAGTCGTGTGTTCACAGAGACCTGGCCGCCAGGAACGTGCTTGTC
ACCCACGGGAAAGTGGTGAAGATATGTGACTTTGGATTGGCTCGAGATATCATGAGTGATTCCAACTATGTTGTCAGGGGCAATGCC
CGTCTGCCTGTAAAATGGATGGCCCCCGAAAGCCTGTTTGAAGGCATCTACACCATTAAGAGTGATGTCTGGTCATATGGAATATTA
CTGTGGGAAATCTTCTCACTTGGTGTGAATCCTTACCCTGGCATTCCGGTTGATGCTAACTTCTACAAACTGATTCAAAATGGATTT
AAAATGGATCAGCCATTTTATGCTACAGAAGAAATATACATTATAATGCAATCCTGCTGGGCTTTTGACTCAAGGAAACGGCCATCC
TTCCCTAATTTGACTTCGTTTTTAGGATGTCAGCTGGCAGATGCAGAAGAAGCGATGTATCAGAATGTGGATGGCCGTGTTTCGGAA
TGTCCTCACACCTACCAAAACAGGCGACCTTTCAGCAGAGAGATGGATTTGGGGCTACTCTCTCCGCAGGCTCAGGTCGAAGATTCG
TAGAGGAACAATTTAGTTTTAAGGACTTCATCCCTCCACCTATCCCTAACAGGCTGTAGATTACCAAAACAAGATTAATTTCATCAC
TAAAAGAAAATCTATTATCAACTGCTGCTTCACCAGACTTTTCTCTAGAAGCTGTCTGCGTTTACTCTTGTTTTCAAAGGGACTTTT
GTAAAATCAAATCATCCTGTCACAAGGCAGGAGGAGCTGATAATGAACTTTATTGGAGCATTGATCTGCATCCAAGGCCTTCTCAGG
CTGGCTTGAGTGAATTGTGTACCTGAAGTACAGTATATTCTTGTAAATACATAAAACAAAAGCATTTTGCTAAGGAGAAGCTAATAT
GATTTTTTAAGTCTATGTTTTAAAATAATATGTAAATTTTTCAGCTATTTAGTGATATATTTTATGGGTGGAATAAAATTTCTA**CT**
ACAGAATTGCCCATTATTGAATTATTTACATGGTATAATTAGGGCAAGTCTTAACTGGAGTTCACGAACCCCCTGAAATTGTGCACC
CATAGCCACCTACACATTCCTTCCAGAGCACGTGTGCTTTTACCCCAAGATACAAGGAATGTGTAGGCAGCTATGGTTGTCACAGCC
TAAGATTTCTGCAACAACAGGGGTTGTATTGGGGGAAGTTTATAATGAATAGGTGTTCTACCATAAAGAGTAATACATCACCTAGAC
ACTTTGGCGGCCTTCCCAGACTCAGGGCCAGTCAGAAGTAACATGGAGGATTAGTATTTTCAATAAAGTTACTCTTGTCCCCACA

Figure S4. mRNA sequence of FLT3. Start and stop codons are colored in blue, 5'UTR is colored in orange and 3'UTR in green. Highlighted in yellow is the canonical sequence for TIS11B binding.

FLT3 amino acid sequence

```
10         20         30         40         50
MPALARDGGQ LPLLVVFSAM IFGTITNQDL PVIKCVLINH KNNDSSVGKS
        60         70         80         90        100
SSYPMVSESP EDLGCALRPQ SSGTVYEAAA VEVDVSASIT LQVLVDAPGN
       110        120        130        140        150
ISCLWVFKHS SLNCQPHFDL QNRGVVSMVI LKMTETQAGE YLLFIQSEAT
       160        170        180        190        200
NYTILFTVSI RNTLLYTLRR PYFRKMENQD ALVCISESVP EPIVEWVLCD
       210        220        230        240        250
SQGESCKEES PAVVKKEEKV LHELFGTDIR CCARNELGRE CTRLFTIDLN
       260        270        280        290        300
QTPQTTLPQL FLKVGEPLWI RCKAVHVNHG FGLTWELENK ALEEGNYFEM
       310        320        330        340        350
STYSTNRTMI RILFAFVSSV ARNDTGYYTC SSSKHPSQSA LVTIVEKGFI
       360        370        380        390        400
NATNSSEDYE IDQYEEFCFS VRFKAYPQIR CTWTFSRKSF PCEQKGLDNG
       410        420        430        440        450
YSISKFCNHK HQPGEYIFHA ENDDAQFTKM FTLNIRRKPQ VLAEASASQA
       460        470        480        490        500
SCFSDGYPLP SWTWKKCSDK SPNCTEEITE GVWNRKANRK VFGQWVSSST
       510        520        530        540        550
LNMSEAIKGF LVKCCAYNSL GTSCETILLN SPGPFPFIQD NISFYATIGV
       560        570        580        590        600
CLLFIVVLTL LICHKYKKQF RYESQLQMVQ VTGSSDNEYF YVDFREYEYD
       610        620        630        640        650
LKWEFPRENL EFGKVLGSGA FGKVMNATAY GISKTGVSIQ VAVKMLKEKA
       660        670        680        690        700
DSSEREALMS ELKMMTQLGS HENIVNLLGA CTLSGPIYLI FEYCCYGDLL
       710        720        730        740        750
NYLRSKREKF HRTWTEIFKE HNFSFYPTFQ SHPNSSMPGS REVQIHPDSD
       760        770        780        790        800
QISGLHGNSF HSEDEIEYEN QKRLEEEEDL NVLTFEDLLC FAYQVAKGME
       810        820        830        840        850
FLEFKSCVHR DLAARNVLVT HGKVVKICDF GLARDIMSDS NYVVRGNARL
       860        870        880        890        900
PVKWMAPESL FEGIYTIKSD VWSYGILLWE IFSLGVNPYP GIPVDANFYK
       910        920        930        940        950
LIQNGFKMDQ PFYATEEIYI IMQSCWAFDS RKRPSFPNLT SFLGCQLADA
       960        970        980        990
EEAMYQNVDG RVSECPHTYQ NRRPFSREMD LGLLSPQAQV EDS
```

Figure S5. FLT3 amino acid sequence. The extracellular domain is highlighted in pink. Transmembrane domain is highlighted in green. Positively charge amino acid from the citosolic domain, indicating potential SET-binding sites are highlighted in yellow and colored. The amino acid implicated in the ITD mutation are highlighted in red

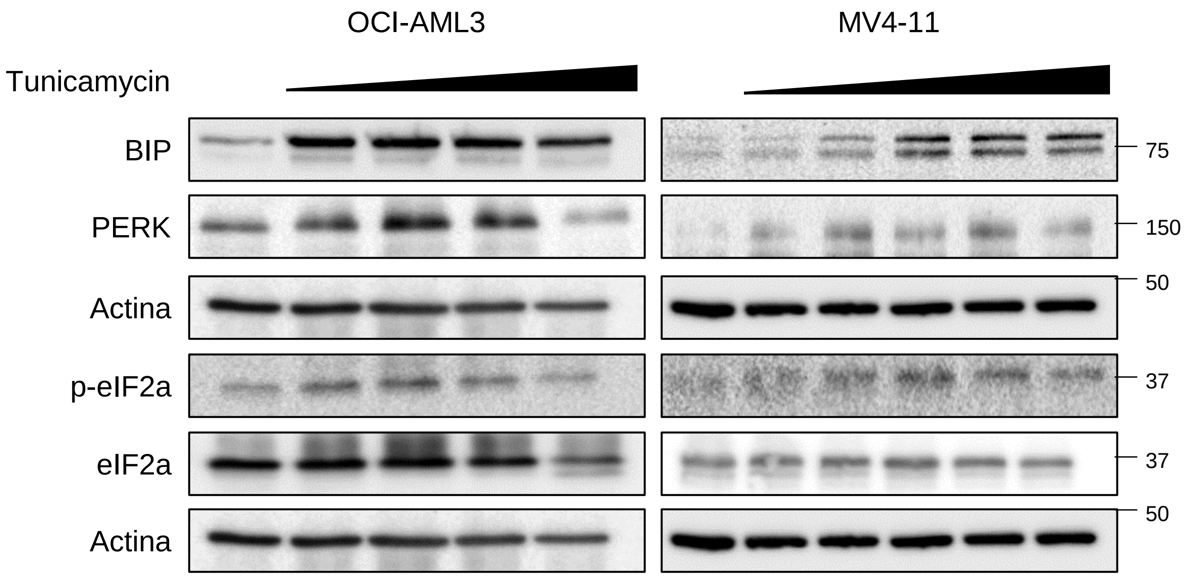

Figure S6.Tunicamcyin induces UPR response. AML cells were treated with increasing concentrations of tunicamycin (0, 25, 50, 100, 200, 400 ng/ ml) for 24 h and western blot analysis of the UPR markers were performed. Representative WB are shown.

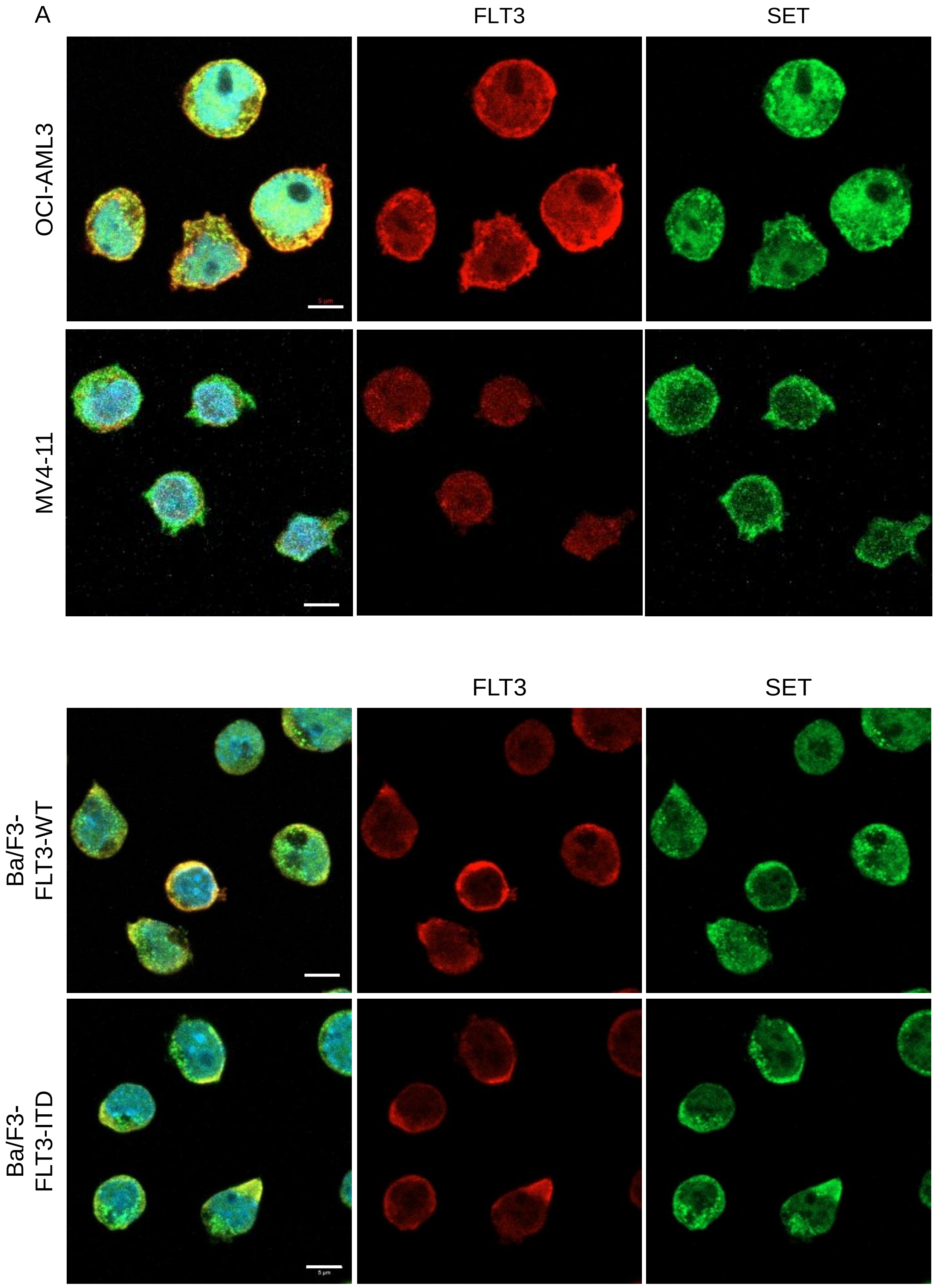
A
FLT3
SET
OCI-AML3
MV4-11
FLT3
SET
Ba/F3-
FLT3-WT
Ba/F3-
FLT3-ITD

B

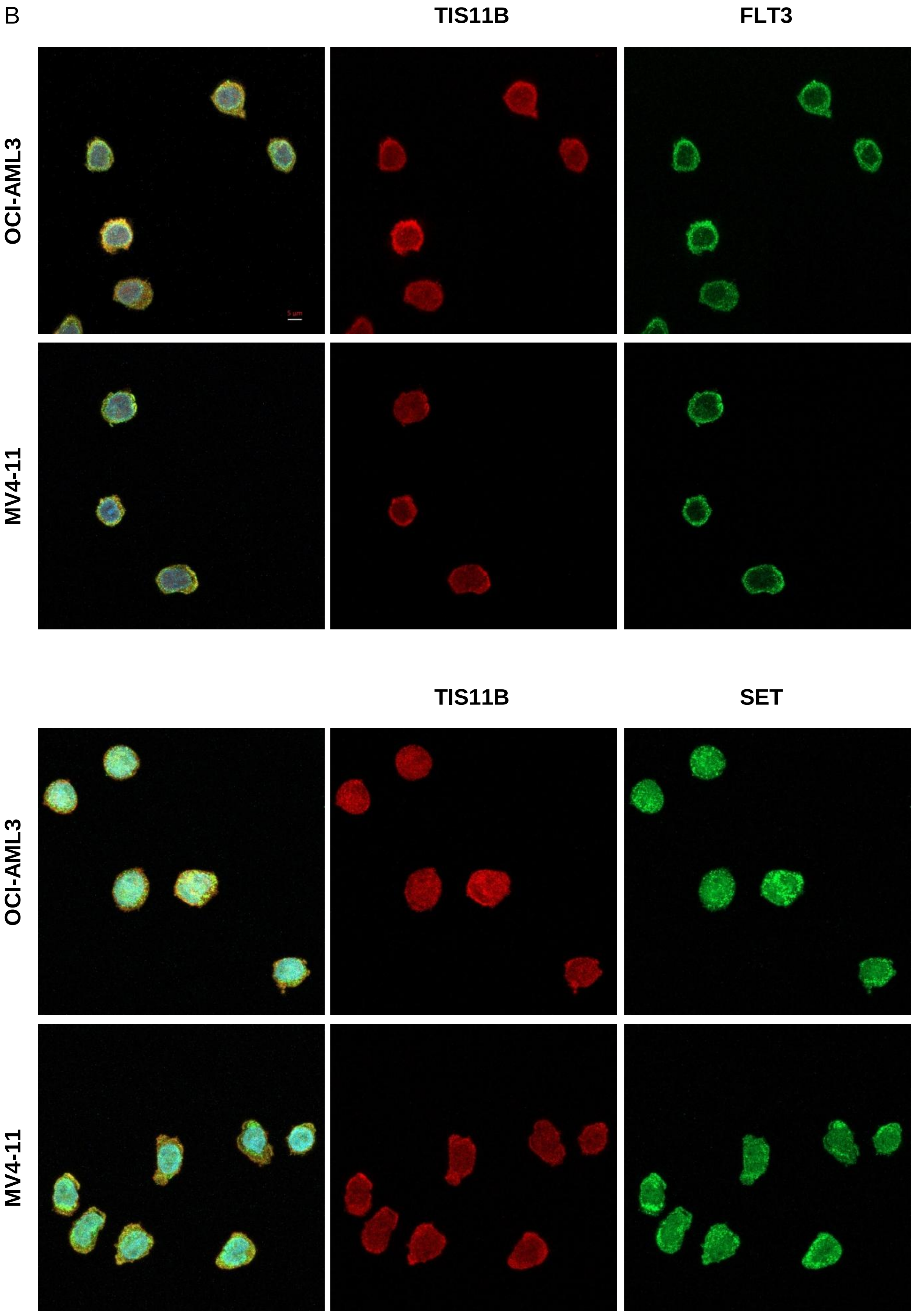

C

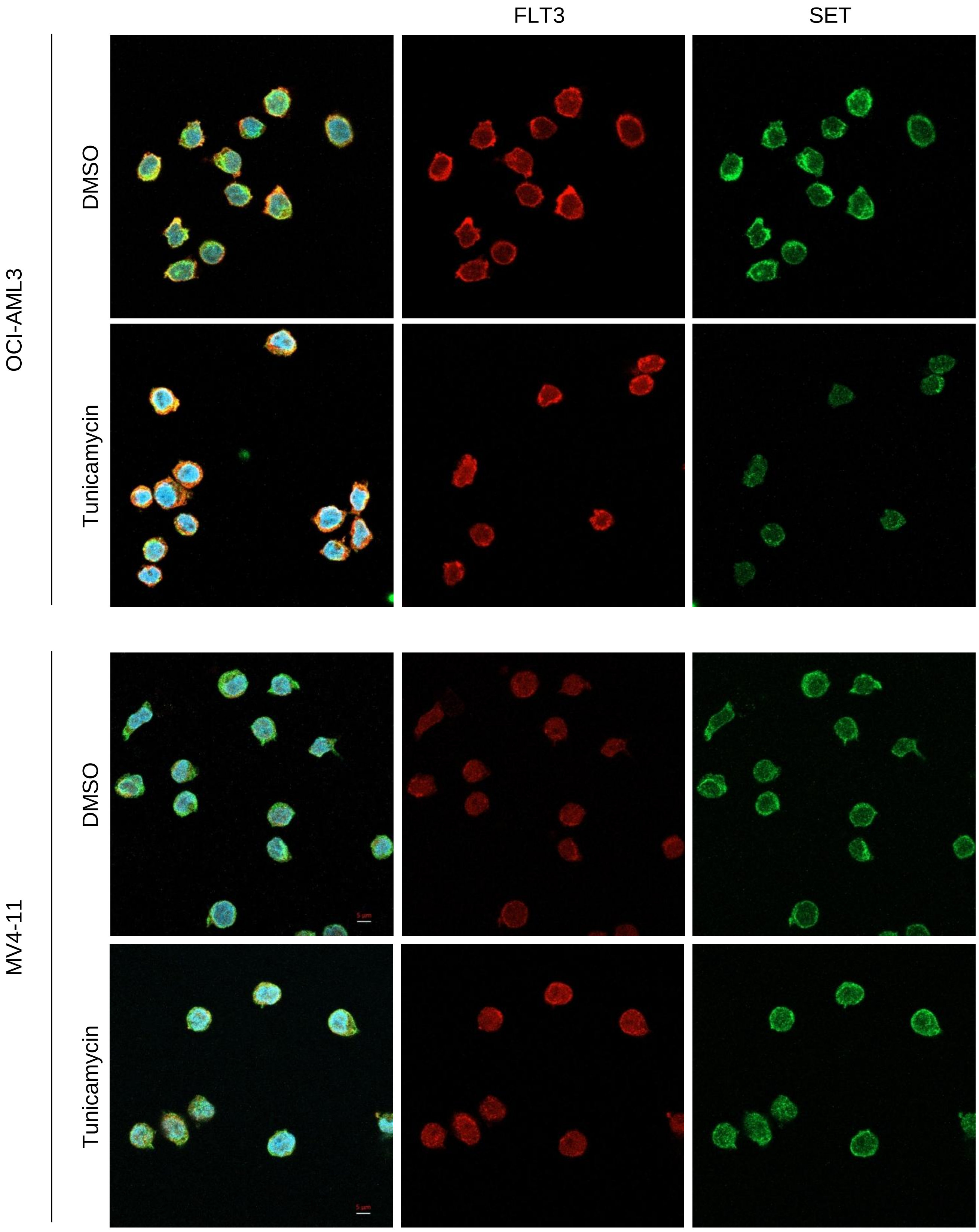

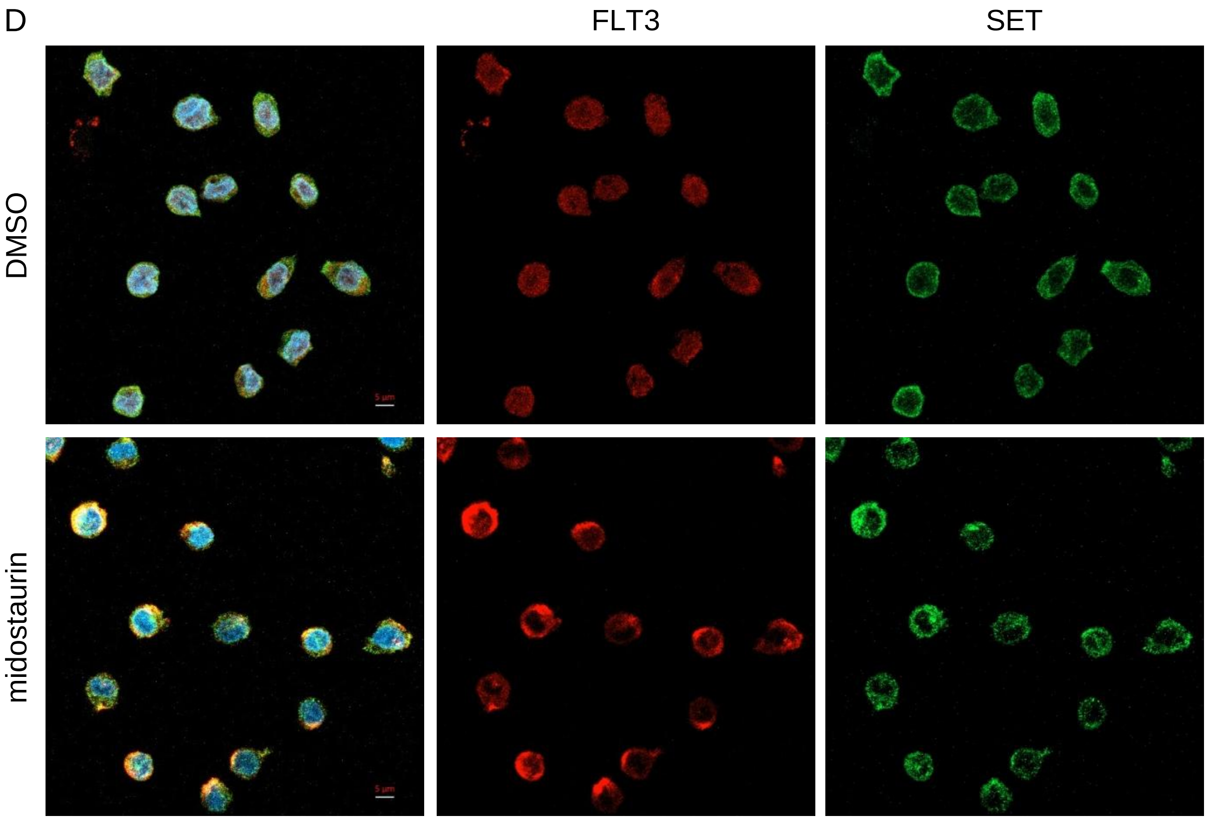

Figure S7. Panoramic view of immunofluorescence. (A) Immunofluorescence of SET (green) and FLT3 (red) in FLT3-WT cell lines (OCI-AML3 and Ba/F3-FLT3-WT) and FLT3-ITD cell lines (MV4-11 and Ba/F3-FLT3-ITD). Confocal microscopy showed co-localization (yellow) of both protein. **(B)** Immunofluorescence of FLT3 (green) and TIS (red) and SET (green) and TIS (red) in FLT3-WT cell line OCI-AML3 and FLT3-ITD cell line MV4-11. Confocal microscopy showed co-localization (yellow) of both protein. **(C)** Immunofluorescence of SET (green) and FLT3 (red) in FLT3-WT cell line OCI-AML3 and FLT3-ITD cell line MV4-11 treated with and without tunicamycin (200 µg/ml) for 24 h. Confocal microscopy showed co-localization (yellow) of both protein. (D) Immunofluorescence of SET (green) and FLT3 (red) in FLT3-WT cell line OCI-AML3 and FLT3-ITD cell line MV4-11 treated with and without midostaurin (20 ng/ml) for 24 h. Confocal microscopy showed co-localization (yellow) of both protein. Nuclei were stained with DAPI (blue). Representative pictures are shown. Immunofluorescences were visualized by confocal microscopy. Experiments were performed in triplicate four times.

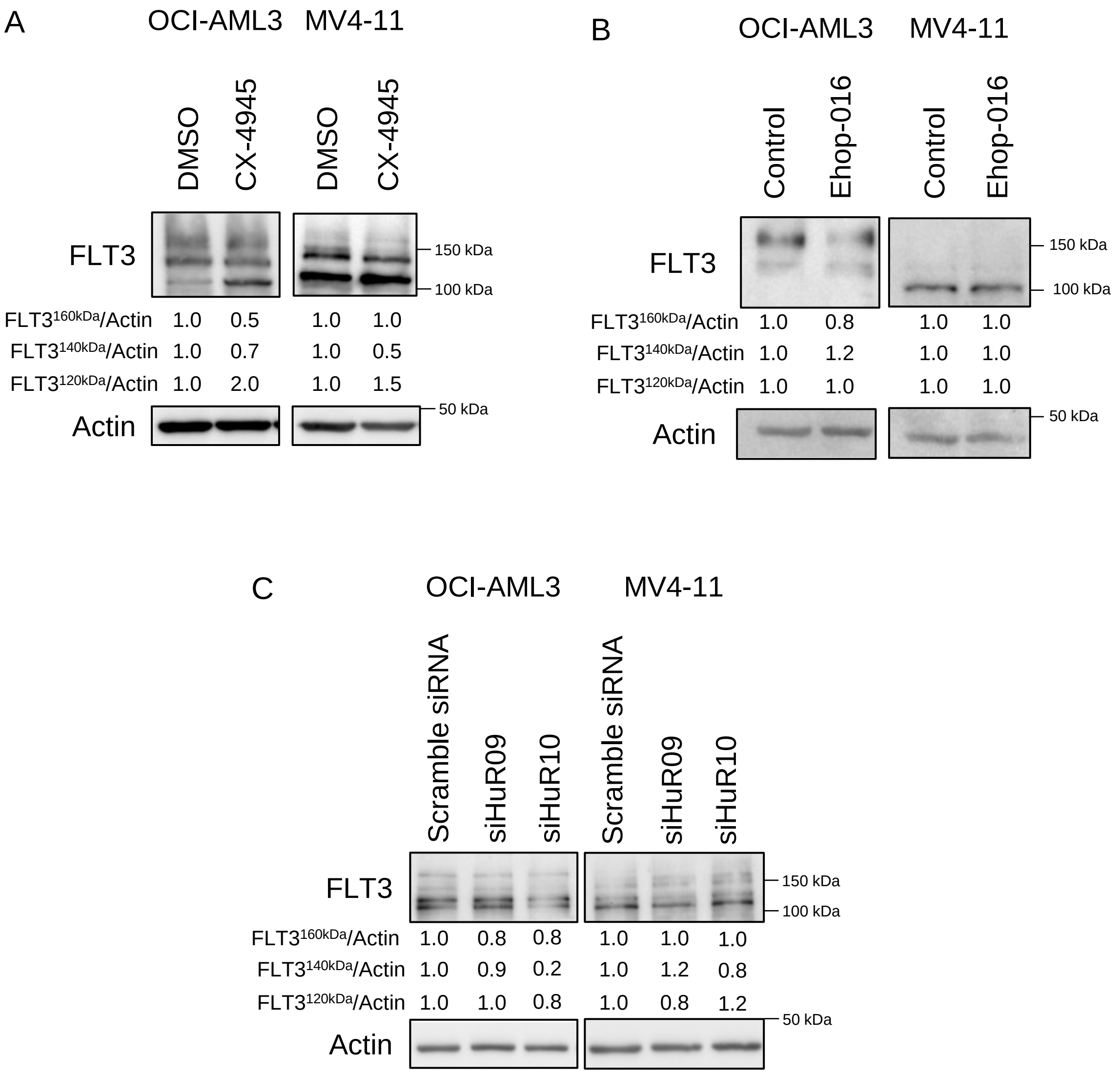

Figure S8. Relative bands of FLT3. Western blot analysis of FLT3 after CX-4945 **(A)** and Ehop-016 treatment **(B)** and HuR silencing **(C)** in OCI-AML and MV4-11 cells. Quantification of the relative FLT3 bands demonstrated a decreased in the 150kDa mature band along with an increase in <150 kDa immature bands in FLT3-WT cells but not in FLT3-ITD cells. A representative WB is shown.

Table S1. List of drugs used

Reagent	Ref#	Source	Concentration
CX-4945	S2248	Selleckchem Chemicals	5µM
Ehop-016	S7319	Selleckchem Chemicals	
FTY720	S5002	Selleckchem Chemicals	5µM
Midostaurin	M1323	SIGMA	20 nM
DT-061	S8774	Selleckchem Chemicals	2.5 and 4 µM

Table S2. List of antibodies used

Target	Ref#	Source	Application
Alexa fluor 488 donkey anti-goat	A-11055	Invitrogen	IF
Alexa fluor 488 donkey anti-mouse	A21202	Invitrogen	IF
Alexa fluor 555 donkey anti-rabbit	A31572	Invitrogen	IF
Alexa fluor 568 donkey anti-mouse	A10037	Invitrogen	IF
Amersham ECL Mouse IgG, HRP-linked whole Ab (from sheep)	NA931	Amersham	WB
Anti-rabbit IgG, HRP-linked Antibody	7074	Cell signaling	WB
Donkey anti-goat IgG HRP affinity purified PAB	04HAF109	R&D systems	WB
PE Mouse anti-Human CD135	558996	BD Pharmingen	FACS
ELAV1/ HuR	12582	cell signalling	WB
ELAV1/ HuR	7468	MERCK	RIP
FLT3	3462	cell signalling	WB, IP
RAC1	66122-1-Ig	Proteintech	WB
SET	A302-261A	Bethyl	IP, WB
SET	sc-5655	Santa Cruz Biotechnology	IF
ZFP36L1/2 (TIS11B	12306-1-AP	Proteintech	IF, WB
β-Actin	A5441	Sigma	WB
