# Supplementary figures and images for: Set Protein Is Involved in FLT3 Membrane Trafficking
Source: Cancers (Basel). 2023 Apr 10;15(8):2233. doi: 10.3390/cancers15082233 (PMC10137103; doi:10.3390/cancers15082233)

Figure 1E

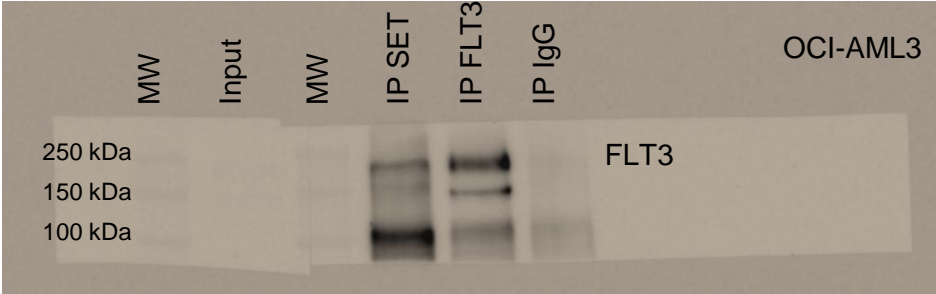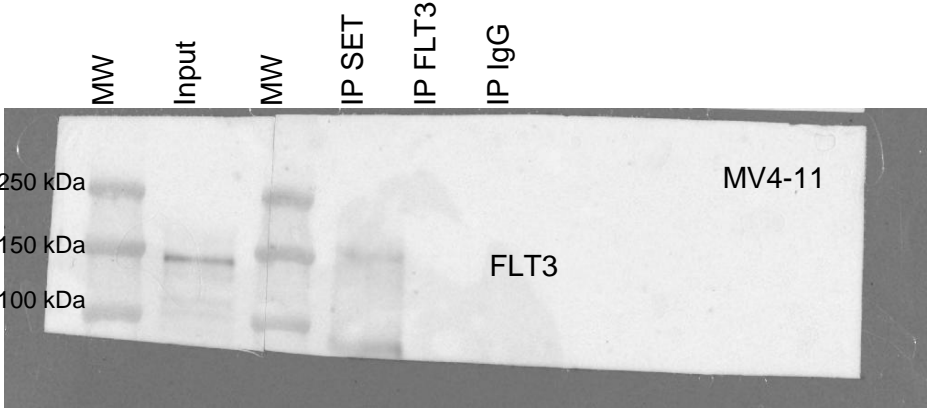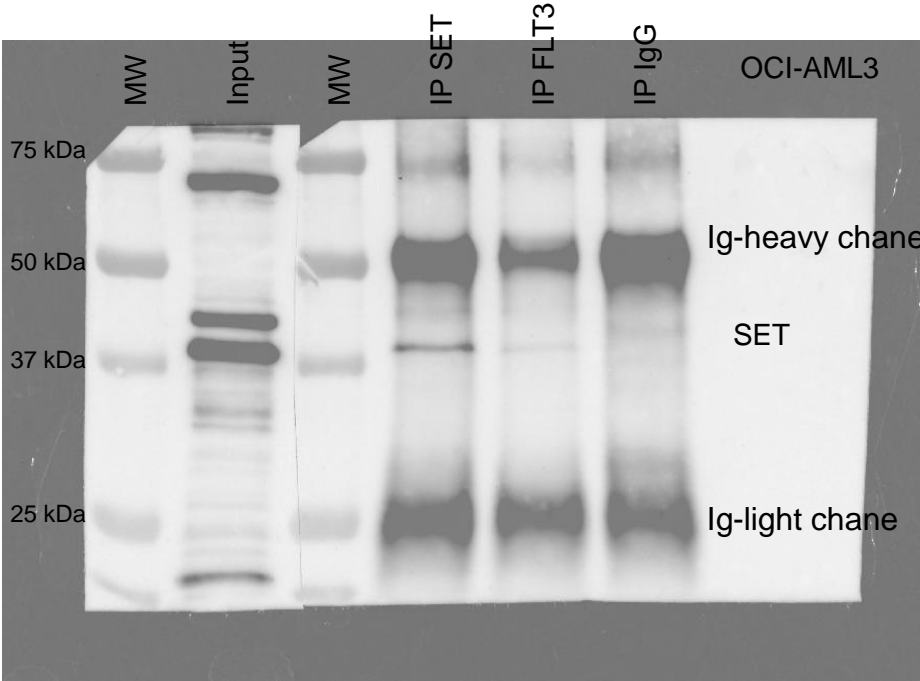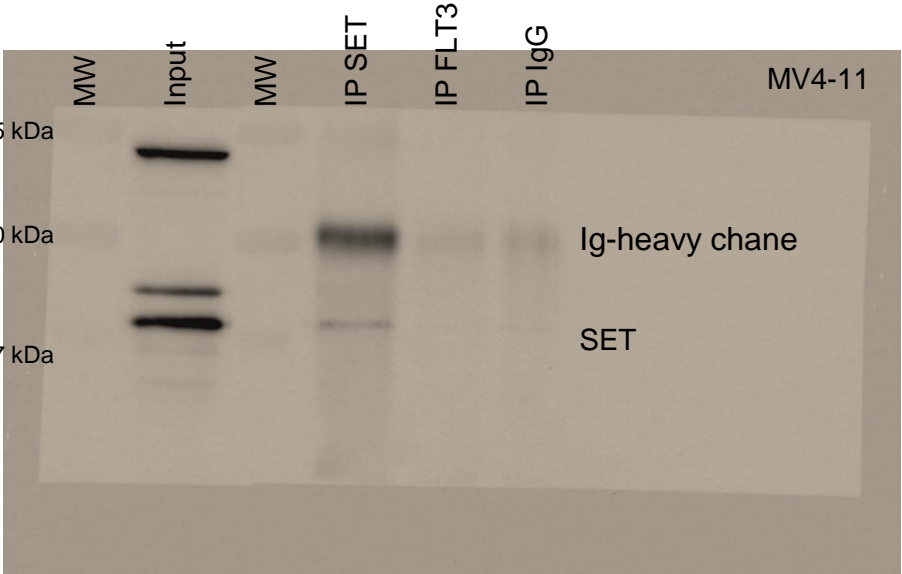

**Figure 2A**

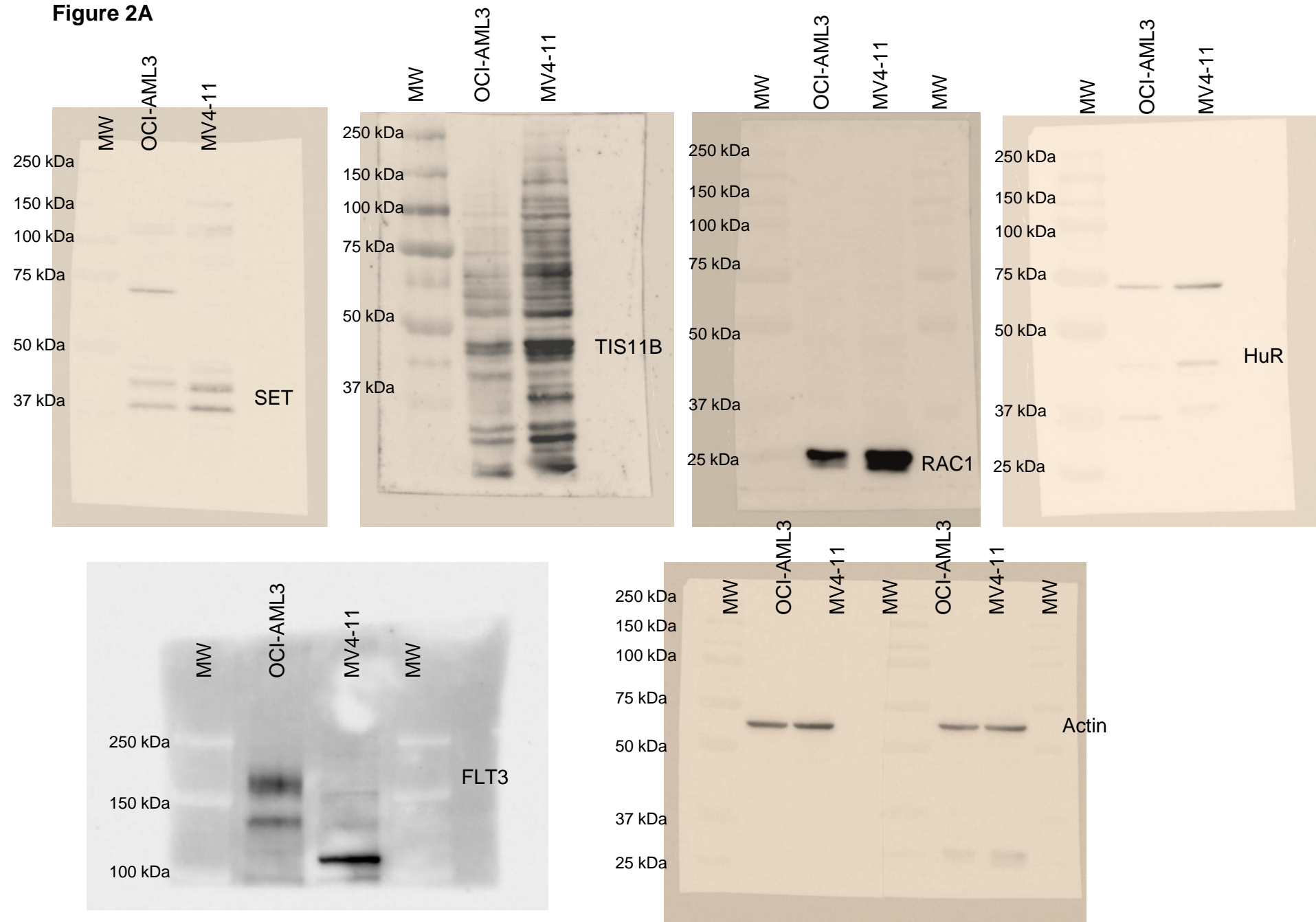

Figure 2E

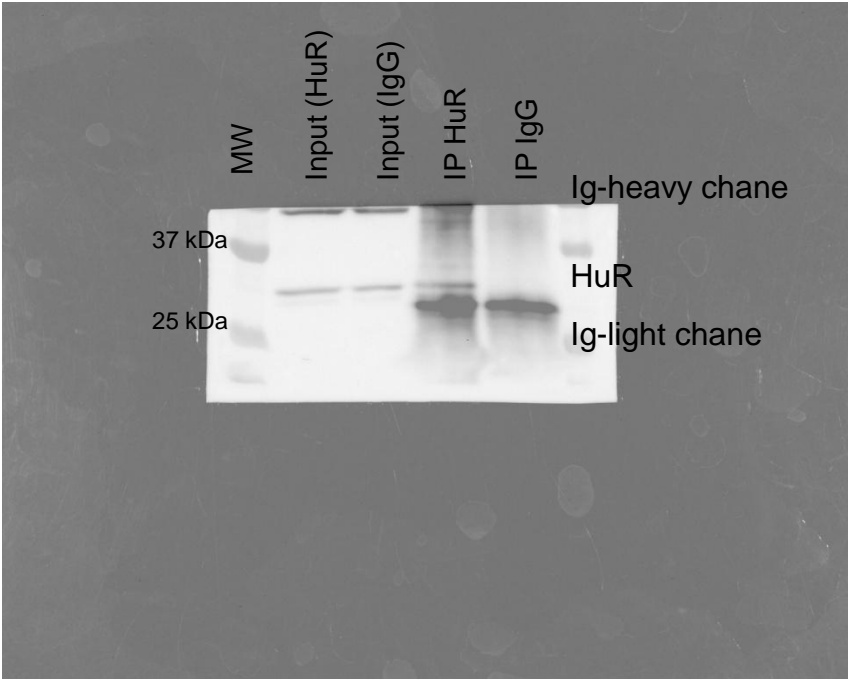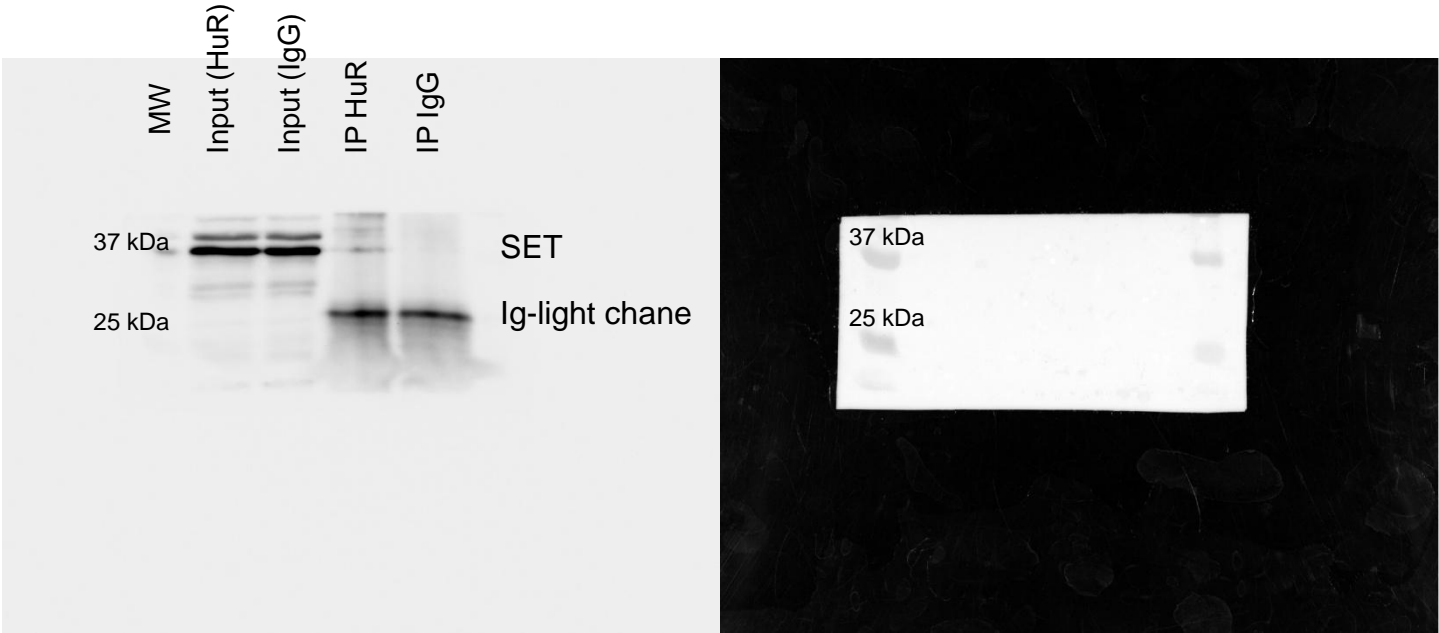

Figure 3C

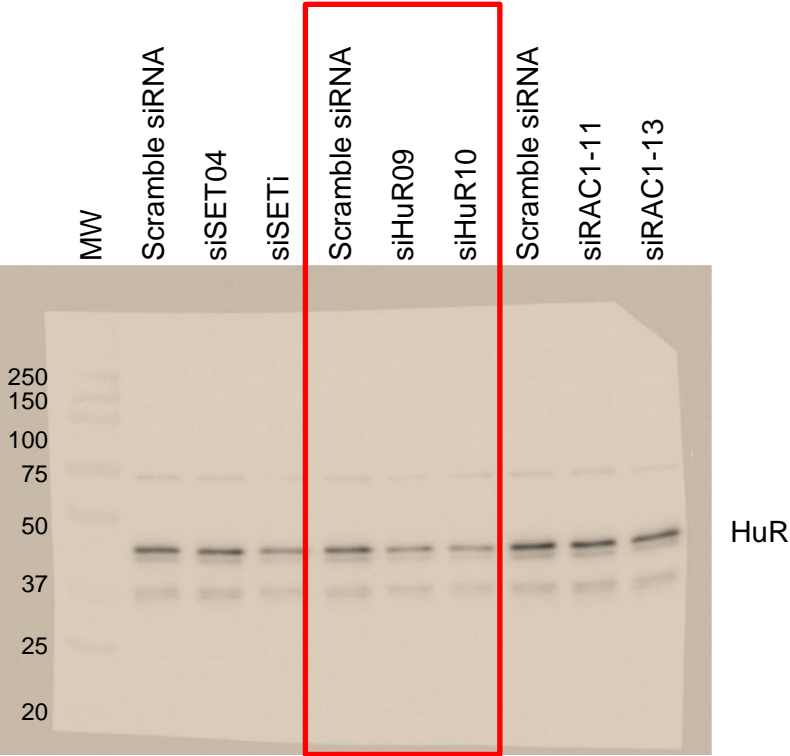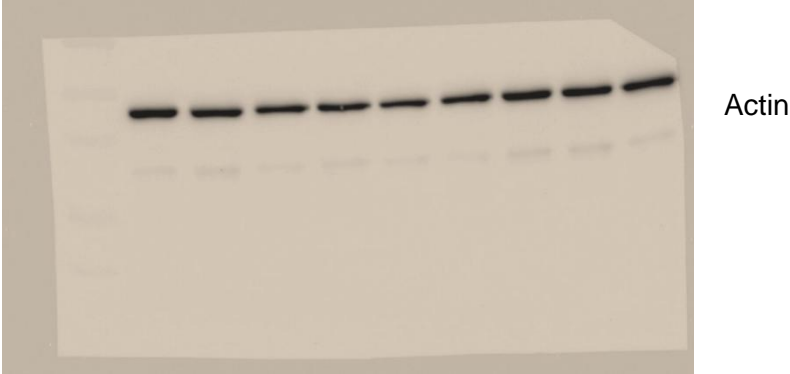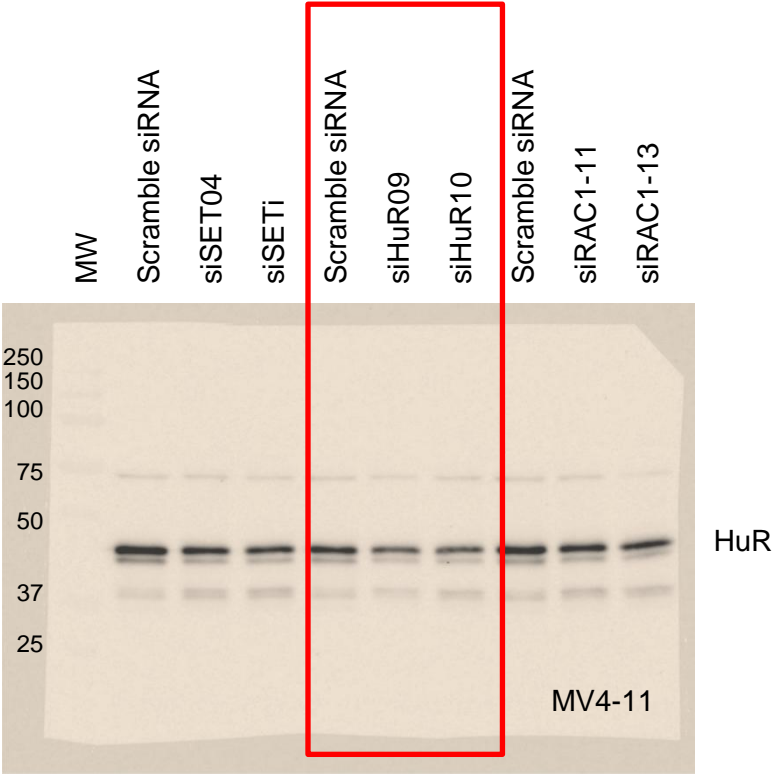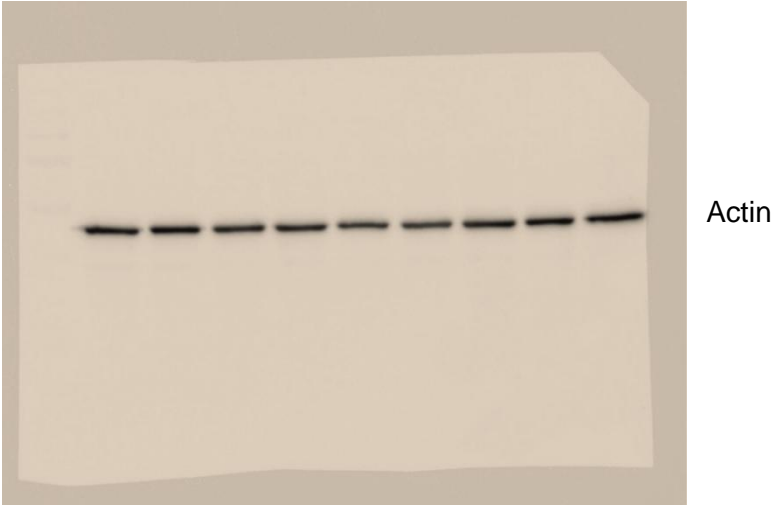

Supplement: Supplementary file 1 [file cancers-15-02233-s001.zip › File S1-Original Images for Blots.pdf]
